# Synthesis and Application of 4′-*C*-[(*N*-alkyl)aminoethyl]thymidine Analogs for Optimizing Oligonucleotide Properties

**DOI:** 10.3390/molecules30030581

**Published:** 2025-01-27

**Authors:** Kota Fujiki, Yuri Kakisawa, Elsayed M. Mahmoud, Yoshihito Ueno

**Affiliations:** 1 Department of Life Science and Chemistry, The Graduate School of Natural Science and Technology, Gifu University, 1-1 Yanagido, Gifu 501-1193, Japan; fujiki.kouta.t9@s.gifu-u.ac.jp; 2 Course of Applied Life Science, Faculty of Applied Biological Sciences, Gifu University, 1-1 Yanagido, Gifu 501-1193, Japanelsayed.maher.mahmoud.saleh.r8@f.gifu-u.ac.jp (E.M.M.); 3 Department of Pharmaceutical Organic Chemistry, Faculty of Pharmacy, Zagazig University, Zagazig 44519, Egypt; 4 United Graduate School of Agricultural Science, Gifu University, 1-1 Yanagido, Gifu 501-1193, Japan; 5 Center for One Medicine Innovative Translational Research (COMIT), Tokai National Higher Education and Research System, Gifu University, 1-1 Yanagido, Gifu 501-1193, Japan

**Keywords:** antisense oligonucleotides, 4′-aminoethyl modification, phosphorothioate, locked nucleic acid, nuclease resistance, RNase H, KRAS, gene silencing

## Abstract

Gapmer-type antisense oligonucleotides (ASOs) are an emerging class of therapeutic agents that directly inhibit pathogenic mRNA. In this study, three new 4′-*C*-substituted thymidine analogs were generated using a synthetic strategy recently established by our group, namely, 4′-*C*-(*N*-ethyl) aminoethyl (4′-EAE-T), 4′-*C*-(*N*-butyl) aminoethyl (4′-BAE-T), and 4′-*C*-(*N*-octyl) aminoethyl (4′-OAE-T). Their properties were evaluated and compared with those of previously reported analogs, including 4′-*C*-aminoethyl (4′-AE-T) and 4′-*C*-(*N*-methyl) aminoethyl (4′-MAE-T). The novel nucleoside analogs were subsequently incorporated into gapmer-type ASOs featuring phosphorothioate (PS) linkages and locked nucleic acids (LNAs) in the wing regions. The incorporation of 4′-EAE-T and 4′-BAE-T analogs resulted in RNA binding affinities similar to that of the previously reported 4′-MAE-T analog, whereas a marked decrease in RNA affinity was noted for 4′-OAE-T, however, this reduction was mitigated when combined with other chemical modifications. Furthermore, the structural modifications conferred enhanced nuclease resistance under bovine serum conditions, with 4′-EAE-T resulting in the highest stability, followed by 4′-BAE-T and 4′-OAE-T. Additionally, oligonucleotides modified with the developed analogs preserved their RNase H cleavage susceptibility, albeit inducing minor alterations in the cleavage pattern. Finally, the oligonucleotides were applied in a gene silencing experiment targeting the KRAS gene, conducted without the use of transfection agents, displaying gene silencing activities comparable to that of the control, with the exception of the 4′-OAE-modified nucleotide, which exhibited low activity.

## 1. Introduction

The use of antisense oligonucleotides (ASOs) for the treatment of diseases that were once considered untreatable by directly targeting the genetic basis of the illness represents a promising therapeutic strategy in modern medicine [1]. These groundbreaking therapeutic agents are single-stranded DNA molecules typically comprising 10–20 bases, designed to hybridize with complementary sequences in mRNA or pre-mRNA, thereby stopping their translation [2]. Upon introduction into the body, ASOs form stable double-stranded structures with their targets, leading to RNase H-mediated degradation of the target mRNA, which lacks a 5′-cap and 3′-polyadenylation structure. This allows for its rapid degradation by intracellular nucleases, resulting in the precise suppression of gene expression [3,4,5,6]. Additionally, ASOs can modulate splicing by binding to pre-mRNA to potentially inhibit gene expression via mechanisms such as exon skipping or inclusion, induced by steric hindrance [7,8].

To realize the full therapeutic potential of ASOs, several challenges must be addressed, such as their rapid degradation by nucleases, and their limited permeability across cell membranes, exacerbated by the repulsive interactions between the membrane lipid bilayer and the ASO phosphodiester backbone, both of which are negatively charged [9]. To overcome these limitations, chemically modified oligonucleotides are being increasingly explored with the aim of maximizing the stability and efficacy [10]. Innovations in nucleotide design, including sugar modifications such as locked nucleic acid (LNA) and phosphorothioate (PS) alterations, have been shown to significantly enhance ASO stability and RNA-binding affinity [11]. Locked nucleic acids (LNAs) are considered one of the most effective modifications. LNAs are chemically constrained nucleotides in which a methylene bridge locks the ribose sugar into a non-flexible conformation. This structural rigidity increases the hybridization affinity of ASOs for their RNA targets, allowing shorter oligonucleotides with superior binding specificity and resistance to degradation. The high thermal stability conferred by LNAs makes them particularly well-suited for the wing regions of ASOs, where strong binding to target sequences is critical for therapeutic efficacy [12]. Phosphorothioate (PS) modifications represent another crucial advancement in ASO design. These involve exchanging a non-bridging oxygen atom in the phosphate group with sulfur, which significantly enhances nuclease resistance and facilitates cellular uptake [13,14,15]. However, despite their extensive use in clinical applications, PS modifications have been associated with cytotoxicity concerns, requiring further optimization [16,17]. Gapmer-type ASOs comprising a natural-DNA central region and RNA-modified wings at both ends enable the leveraging of LNAs to achieve superior target affinity and protection against degradation [18,19,20,21,22]. The natural DNA core ensures recognition by RNase H, while PS modifications increase endonuclease resistance.

Recent research has focused on further refining ASO designs by incorporating novel chemical modifications. For instance, Wang et al. and Ueno et al. synthesized 4′-aminoalkylthymidines and evaluated their structure-activity relationships [23,24]. The authors described that the cationic nature of the aminoalkyl groups conferred enhanced nuclease resistance through electrostatic repulsion, while it did not affect recognition by RNase H. Interestingly, subsequent studies indicated that lengthening the side chains of these analogs can further increase nuclease resistance, although occasionally at the expense of RNA affinity [25]. It has also been revealed that extending the 2′-*O*-side chain leads to increased nuclease resistance [26]. Collectively, this suggests that side chain extension constitutes an effective design strategy for improving nuclease resistance.

**Figure 1:**
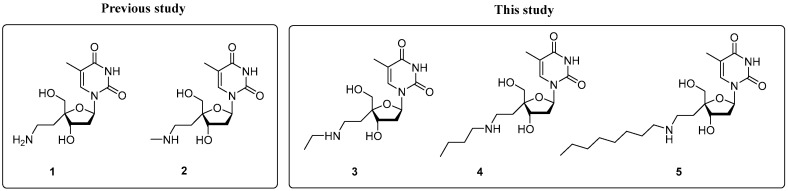
The chemical structures of the previously developed thymidine derivatives, 4′-AE-T (**1**), 4′-MAE-T (**2**), serve as reference points for this study. In comparison, newly synthesized thymidine derivatives introduced and investigated here include 4′-EAE-T (**3**), 4′-BAE-T (**4**), and 4′-OAE-T (**5**).

Building on this foundation, our group recently reported the synthesis of 4′-*C*-(*N*-methyl)aminoethylthymidine, which demonstrated improved nuclease resistance with sustained RNase H activity [27]. Notably, the introduction of lipophilic substituents has been shown to improve antisense activity and modify internal dynamics, therefore, this design strategy was implemented in this work. Based on the insights from previous studies, we proposed that modifying oligonucleotides by integrating longer alkyl chains alongside the positively charged amino group in the aminoethyl moiety could significantly enhance their functional properties. This approach exploits the dual role of the amino group, which not only offsets the negative charge of oligonucleotides but also facilitates their interaction with negatively charged cell membranes. These structural alterations are expected to optimize oligonucleotide stability, improve cellular uptake, and enhance overall efficacy. Herein, we applied our previously developed synthetic route for accessing the 4′-aminoethyl framework to generate three novel 4′-modified nucleotides, namely 4′-*C*-(*N*-ethyl)aminoethyl (4′-EAE-T) (**3**), 4′-*C*-(*N*-butyl)aminoethyl (4′-BAE-T) (**4**), and 4′-*C*-(*N*-octyl)aminoethyl (4′-OAE-T) (**5**) (Figure 1) with the aim of enhancing their lipid solubility and basicity, and in turn, their nuclease resistance and cellular uptake.

To evaluate the impact of these modifications, we synthesized ASO/RNAs containing the novel analogs, assessed their fundamental properties, and compared them with those containing previously reported 4′-AE-T and 4′-MAE-T units. Thus, we synthesized gapmer-type ASOs containing LNA regions in the wings and 4′-alkylaminoethylthymidines in the gap regions, as well as a PS-modified phosphate backbone to investigate their efficacy in suppressing KRAS gene expression in cellular models. Overall, the findings of this study will contribute to the optimization of nucleotide designs intended for therapeutic applications.

## 2. Results and Discussion

### 2.1. Synthesis of Nucleoside Analogs

**Scheme 1:**
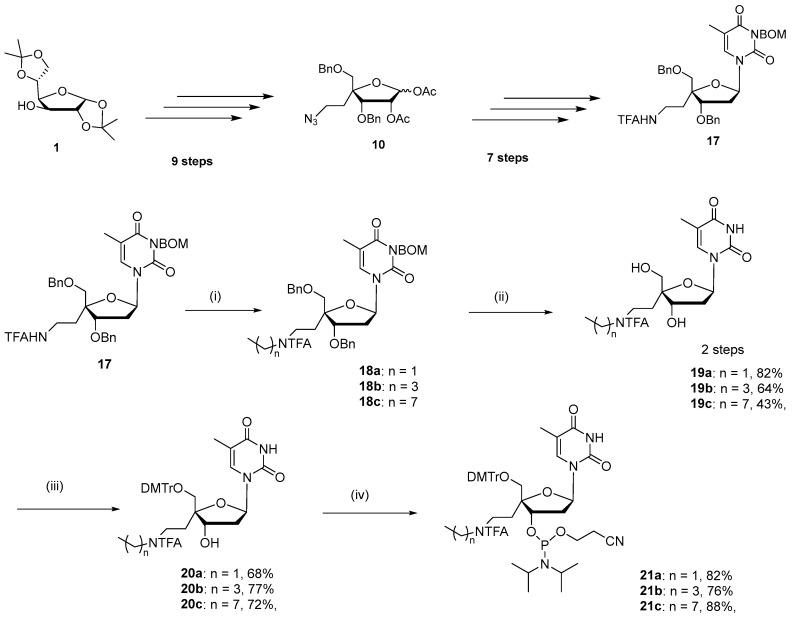
Synthesis of 4′-*C*-(2-*N*-ethyl)aminoethylthymidine (4′-EAE-T), 4′-*C*-(2-*N*-butyl)aminoethylthymidine (4′-BAE-T), and 4′-*C*-(2-*N*-octyl)aminoethylthymidine (4′-OAE-T) phosphoramidites **21a**–**c** ^a^. ^a^ Reagents and conditions: (i) NaH, alkyl iodide, DMF, r.t., 3 h; (ii) BCl_3_, CH_2_Cl_2_, −78 °C to −30 °C, 5 h; (iii) DMTrCl, DMAP, pyridine, r. t., 23 h; (iv) 2-cyanoethyl *N,N*-diisopropylchlorophosphoramidite, *N, N*-diisopropylethylamine (DIPEA), THF, r.t., 1.5 h.

The synthetic methodology employed for accessing the target phosphoramidites (**21a**–**c**) is depicted in Scheme 1. Starting from diacetone-D-glucose (**1**), 4′,5′-di-*O*-benzyl-3-*N*-benzyloxymethyl-4′-*C*-(2-trifluoroacetylaminoethyl)thymidine (**17**) was obtained via our previously established multi-step procedure [27]. Thus, **1** was subjected to a multi-step reaction sequence to form intermediate **10**, which was subsequently transformed into compound **17**, the key intermediate for the synthesis of the desired analogs. The reaction of **17** with ethyl, butyl, and octyl iodides in the presence of NaH afforded compounds **18a**–**c**, which were used in the subsequent steps without further purification. These products were further treated with BCl_3_ to deprotect the 4′,5′-dibenzyl and the 3-benzyloxymethyl groups, affording the corresponding deprotected products **19a**–**c** in yields ranging from 43% to 82%. The 5′-hydroxy groups of **19a**–**c** were subsequently protected with 4,4′-dimethoxytrityl (DMTr), delivering compounds **20a**–**c** in yields of 68–77%. The final step involved the phosphitylation of **20a**–**c** to afford the desired phosphoramidites **21a**–**c** in 76–88% yield.

### 2.2. Synthesis of Oligonucleotides

DNA oligomers containing 4′-EAE-T, 4′-BAE-T or 4′-OAE-T analogs were synthesized via the solid-phase phosphoramidite method using a DNA/RNA automatic synthesizer. After synthesis, the DNA oligomers were treated with aq. NH_3_ for 12 h at 55 °C to cleave the CPG bead and remove the acyl groups. The cyanoethyl group was selectively removed from oligomers containing 4′-EAE-T, 4′-BAE-T or 4′-OAE-T via treatment with 10% diethylamine in MeCN for 5 min before NH_3_ treatment. For the LNA gapmers, universal supports were used instead of dT-CPG, cleavage and deprotection were performed using the above-described procedure. RNA oligomers were cleaved from CPG beads and deprotected via treatment with concentrated NH_3_ solution/40% methylamine (1:1, *v*/*v*) for 10 min at 65 °C. Subsequently, deprotection of the 2′-O-TBDMS group was achieved via treatment with Et_3_N·3HF in DMSO for 90 min at 65 °C. The reaction mixture was then desalted using a Sep-Pak C18 cartridge. DNA and RNA oligomers were purified using 20% PolyAcrylamide Gel Electrophoresis (PAGE) and desalted using a Sep-Pak C18 cartridge. The molecular weights of the oligomers were confirmed using MALDI-TOF/MS.

Deoxyoligonucleotides containing analogs **1**, **2**, **3**, **4**, and **5** were successfully synthesized using a DNA/RNA synthesizer according to the phosphoramidite method. The oligonucleotide sequences synthesized in this study are listed in Table 1, Table 2, Table 3, Table 4, Table 5 and Table 6.

### 2.3. Thermal Stability of Duplexes

The effects of 4′-EAE-T, 4′-BAE-T, and 4′-OAE-T modifications on the thermal stability of DNA/RNA heteroduplexes were assessed by measuring melting temperatures (*T*_m_) under conditions where 50% of the duplexes denature (see Figure S1, Supplementary Materials). The *T*_m_ values for the oligonucleotides containing analogs 1 to 5 were compared with those of the native sequence, and the results are shown in Table 1.

We previously reported that the *N*-methylaminoethyl modification decreased the RNA binding affinity to a higher degree than aminoethyl, primarily due to the steric hindrance caused by *N*-methylation [27]. Based on this, it was anticipated that increasing the alkyl chain length of the aminoethyl segment to ethyl, butyl, or octyl would further decrease RNA affinity. However, contrary to this speculation, the present findings revealed no significant decline in the melting temperature of the nucleotides containing longer alkyl chains.

The results showed that the incorporation of aminoalkyl-modified nucleotides generally resulted in slightly lower RNA affinities compared to that of the native duplex. For example, the *T*_m_ of duplex **2**, modified with 4′-AE-T, decreased by 2.36 °C relative to that of duplex **1**, corresponding to a decrease of 0.59 °C per modification. Similarly, the insertion of 4′-MAE-T analogs in duplex **3** led to a *T*_m_ reduction of 2.83 °C (0.71 °C per modification). Interestingly, the *T*_m_s of duplexes **4** and **5**, modified with 4′-EAE-T and 4′-BAE-T, respectively, decreased by similar amounts (3.06 °C, approximately 0.75 °C per modification), despite the bulkier structure of the butyl group compared to the ethyl group. Conversely, the inclusion of 4′-OAE-T in duplex **6** led to a more pronounced reduction in the *T*_m_ by 6.08 °C (1.52 °C per modification). Altogether, these data suggest that the inclusion of 4′-aminoalkyl groups generally resulted in decreased RNA affinity, with the magnitude depending on the chemical nature of side chain.

The thermal stabilities of the analogs were further examined after their incorporation in gapmer oligonucleotides containing LNA modifications on both wings and PS linkages throughout the backbone (see Figures S2 and S3, Supplementary Materials). The resultant gapmers displayed improved RNA affinities compared to those of previously described duplexes **2**–**6** (Table 2). Although all analog-containing gapmers (duplexes **8**–**17**) displayed slightly lower *T*_m_ values than the natural duplex **7**, the differences were minimal for gapmers containing 4′-AE-T, 4′-MAE-T, 4′-EAE-T, and 4′-BAE-T analogs, ranging from 0.43 to 0.78 °C. In contrast, the decrease in the *T*_m_ of the gapmers comprising the 4′-OAE-T analog at either the 5′ or 3′ end, compared to that of duplex **7**, was the most pronounced with a magnitude of 1.44 and 2.04 °C, respectively. Despite the reduction in the *T*_m_, the results suggest that the incorporation of 4′-OAE-T analogs in combination with other chemical modifications does not significantly alter gapmer ASO activity.

To further elucidate the observed differences in the *T*_m_, the thermodynamic parameters of duplexes **1**–**6** were determined using the slope of the plot of 1/*T*_m_ versus (C_T_/4), where C_T_ is the total concentration of ssDNA; the results are summarized in Table 3. The standard Gibbs free energy change (ΔG°) for the unmodified duplex **1** was −17.1 kcal/mol at 37 °C, whereas the corresponding values for duplexes **2**–**6** were −16.5, −15.6, −17.1, −17.1, and −13.2 kcal/mol, respectively. The enthalpy change (ΔH°) values for duplexes **1**–**6** were −130.8, −129.9, −116.3, −143.0, −146.2, and −90.8 kcal/mol, with corresponding entropy changes (ΔS°) of −366.6, −365.9, −324.6, −406.1, −414.7, and −250.2 kcal/mol, respectively. These results indicate that for duplexes containing 4′-AE-T and 4′-MAE-T, the slight thermal destabilization observed following their incorporation is attributable to unfavorable enthalpy changes. In contrast, duplexes with 4′-EAE-T and 4′-BAE-T modifications demonstrated superior thermodynamic stability, possibly due to stronger enthalpic interactions. On the other hand, the substantial destabilization of duplex **6**, modified with 4′-OAE-T, likely originates from a combination of unfavorable enthalpy and favorable entropy changes, suggesting hydrophobic interactions among the *N*-(octyl)aminoethyl side chains.

**Table 1:** Sequence of DNA/RNA duplexes, DNAs, and RNAs, and the *T*_m_ values of the duplexes.

Abbreviation of Duplex	Abbreviation of DNA and RNA	Sequence ^a^	*T*_m_ (°C) ^b^	Δ*T*_m_ (°C) ^c^	Δ*T*_m_ (°C)/mod.
Duplex **1**	DNA **1**	5′-d(TCTTTCTCTTTCCCTT)-3′	59.6 (±0.08)	–	–
	RNA **1**	3′-r(AAGGGAAAGAGAAAGA)-5′			
Duplex **2**	DNA **2**	5′-d(TCTTTCTCTTTCCCTT)-3′	57.3 (±0.21)	−2.36	−0.59
	RNA **1**	3′-r(AAGGGAAAGAGAAAGA)-5′			
Duplex **3**	DNA **3**	5′-d(TCTTTCTCTTTCCCTT)-3′	56.8 (±0.21)	−2.83	−0.71
	RNA **1**	3′-r(AAGGGAAAGAGAAAGA)-5′			
Duplex **4**	DNA **4**	5′-d(TCTTTCTCTTTCCCTT)-3′	56.6 (±0.08)	−3.06	−0.76
	RNA **1**	3′-r(AAGGGAAAGAGAAAGA)-5′			
Duplex **5**	DNA **5**	5′-d(TCTTTCTCTTTCCCTT)-3′	56.7 (±0.0.06)	−3.00	−0.75
	RNA **1**	3′-r(AAGGGAAAGAGAAAGA)-5′			
Duplex **6**	DNA **6**	5′-d(TCTTTCTCTTTCCCTT)-3′	53.6 (±0.01)	−6.08	−1.52
	RNA **1**	3′-r(AAGGGAAAGAGAAAGA)-5′			

^a^ red, blue, yellow, green and purple denote 4′-AE-T (analog **1**), 4′-MAE-T (analog **2**), 4′-EAE-T (analog **3**), 4′-BAE-T (analog **4**) and 4′-OAE-T (analog **5**), respectively. ^b^ The *T*_m_s were measured in a buffer containing 10 mM sodium phosphate (pH 7.0) and 100 mM NaCl. The concentration of duplexes was 3 µM. All experiments were carried out three times, and data are presented as the mean ± SD. ^c^ Δ*T*_m_ = [*T*_m_ (Duplex_mod_) − *T*_m_ (Duplex **1**)].

**Table 2:** Sequence of gapmer-type ASO, DNAs and RNAs, and the *T*_m_ values of duplexes.

Abbreviation of Duplex	Abbreviation of DNA and RNA	Sequence ^a^	*T*_m_ (°C) ^b^	Δ*T*_m_ (°C) ^c^
Duplex **7**	DNA **13**	5′-(G^C^T^d(A^T^T^A^G^G^A^G^T^C^T^T^T)-3′	62.0 (±0.08)	–
	RNA **2**	3′-r(AAAGACUCCUAAUAGC)-5′		
Duplex **8**	DNA **14**	5′-(G^C^T^d(A^T^T^A^G^G^A^G^T^C)^T^T^T)-3′	61.5 (±0.17)	−0.54
	RNA **2**	3′-r(AAAGACUCCUAAUAGC)-5′		
Duplex **9**	DNA **15**	5′-(G^C^T^d(A^T^T^A^G^G^A^G^T^C)^T^T^T)-3′	61.4 (±0.23)	−0.64
	RNA **2**	3′-r(AAAGACUCCUAAUAGC)-5′		
Duplex **10**	DNA **16**	5′-(G^C^T^d(A^T^T^A^G^G^A^G^T^C)^T^T^T)-3′	61.5 (±0.09)	−0.53
	RNA **2**	3′-r(AAAGACUCCUAAUAGC-5′		
Duplex **11**	DNA **17**	5′-(G^C^T^d(A^T^T^A^G^G^A^G^T^C)^T^T^T)-3′	61.6 (±0.14)	−0.45
	RNA **2**	3′-r(AAAGACUCCUAAUAGC)-5′		
Duplex **12**	DNA **18**	5′-G^C^T^d(A^T^T^A^G^G^A^G^T^C)^T^T^T-3′	61.5(±0.09)	−0.53
	RNA **2**	3′-r(AAAGACUCCUAAUAGC)-5′		
Duplex **13**	DNA **19**	5′-G^C^T^d(A^T^T^A^G^G^A^G^T^C)^T^C^T-3′	61.4 (±0.22)	−0.64
	RNA **2**	3′-r(AAAGACUCCUAAUAGC)-5′		
Duplex **14**	DNA **20**	5′-G^C^T^d(A^T^T^A^G^G^A^G^T^C)^T^T^T-3′	61.6 (±0.14)	−0.43
	RNA **2**	3′-r(AAAGACUCCUAAUAGC)-5′		
Duplex **15**	DNA **21**	5′-G^C^T^d(A^T^T^A^G^G^A^G^T^C)^T^T^T-3′	61.2 (±0.12)	−0.78
	RNA **2**	3′-r(AAAGACUCCUAAUAGC-5′		
Duplex **16**	DNA **22**	5′-G^C^T^d(A^T^T^A^G^G^A^G^T^C)^T^T^T-3′	60.6 (±0.18)	−1.44
	RNA **2**	3′-r(AAAGACUCCUAAUAGC)-5′		
Duplex **17**	DNA **23**	5′-G^C^T^d(A^T^T^A^G^G^A^G^T^C)^T^T^T-3′	59.9 (±0.22)	−2.04
	RNA **2**	3′-r(AAAGACUCCUAAUAGC)-5′		

^a^ red, blue, yellow, green, and purple denote 4′-AE-T (analog **1**), 4′-MAE-T (analog **2**), 4′-EAE-T (analog **3**), 4′-BAE-T (analog **4**) and 4′-OAE-T (analog **5**), respectively. ^ and underline denote phosphorothioate (PS) linkage and locked nucleic acids (LNA), respectively. ^b^ The *T*_m_s were measured in a buffer containing 10 mM sodium phosphate (pH 7.0) and 100 mM NaCl. The concentration of duplexes was 3 µM. All experiments were carried out three times, and data are presented as the mean ± SD. ^c^ Δ*T*_m_ = [*T*_m_ (Duplex_mod_) − *T*_m_ (Duplex **7**)]. The chemical structure of LNA = 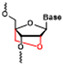.

**Table 3:** Thermodynamic parameters of the DNA/RNA modified duplexes.

Abbreviation of Duplex	Abbreviation of RNA	Sequence ^a^	*T*_m_(°C) ^b^	Δ*T*_m_ (°C)/mod. ^c^	Δ*H°* (kcal/mol) ^d^	Δ*S°* (cal/mol·K) ^d^	Δ*G°*_37°C_ (kcal/mol) ^d^
Duplex **1**	DNA **1**	5′-d(TCTTTCTCTTTCCCTT)-3′	59.6 (±0.08)	–	−130.8	−366.6	−17.1
	RNA **1**	3′-r(AAGGGAAAGAGAAAGA)-5′					
Duplex **2**	DNA **2**	5′-d(TCTTTCTCTTTCCCTT)-3′	57.3 (±0.21)	−0.59	−129.9	−365.9	−16.5
	RNA **1**	3′-r(AAGGGAAAGAGAAAGA)-5′					
Duplex **3**	DNA **3**	5′-d(TCTTTCTCTTTCCCTT)-3′	56.8 (±0.21)	−0.71	−116.3	−324.6	−15.6
	RNA **1**	3′-r(AAGGGAAAGAGAAAGA)-5′					
Duplex **4**	DNA **4**	5′-d(TCTTTCTCTTTCCCTT)-3′	56.6 (±0.08)	−0.76	−143.0	−406.1	−17.1
	RNA **1**	3′-r(AAGGGAAAGAGAAAGA)-5′					
Duplex **5**	DNA **5**	5′-d(TCTTTCTCTTTCCCTT)-3′	56.7 (±0.06)	−0.75	−146.2	−414.7	−17.6
	RNA **1**	3′-r(AAGGGAAAGAGAAAGA)-5′					
Duplex **6**	DNA **6**	5′-d(TCTTTCTCTTTCCCTT)-3′	53.6 (±0.01)	−1.52	−90.8	−250.2	−13.2
	RNA **1**	3′-r(AAGGGAAAGAGAAAGA)-5′					

^a^ red, blue, yellow, green, and purple denote 4′-AE-T (analog **1**), 4′-MAE-T (analog **2**), 4′-EAE-T (analog **3**), 4′-BAE-T (analog **4**) and 4′-OAE-T (analog **5**), respectively. ^b^ The *T*_m_s were measured in a buffer containing 10 mM sodium phosphate (pH 7.0) and 100 mM NaCl. The concentration of duplexes was 3 µM. All experiments were carried out three times, and data are presented as the mean ± SD. ^c^ Δ*T*_m_ = [*T*_m_ (Duplex_mod_) − *T*_m_ (Duplex **1**)]. ^d^ Thermodynamic parameters were determined by plotting 1/*T*_m_ against C_T_/4.

### 2.4. CD Spectra

**Figure 2:**
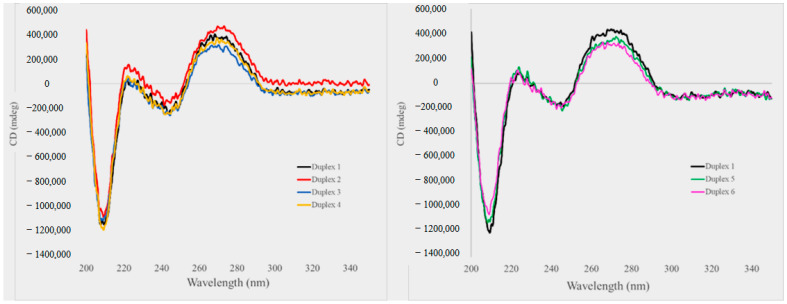
CD spectra of the native and modified DNA/RNA duplexes **1**–**6** in a buffer containing 10 mM sodium phosphate (pH 7.0) and 100 nM NaCl at 25 °C. The concentration of the duplexes was 4 μM. Duplexes **1**, **2**, **3**, **4**, **5** and **6** denote unmodified, 4′-AE-T (**1**), 4′-MAE-T (**2**), 4′-EAE-T (**3**), 4′-BAE-T (**4**), and 4′-OAE-T (**5**) analogs, respectively.

Next, circular dichroism (CD) spectra of the natural and chemically modified heteroduplexes were obtained in a 10 mM buffer solution to evaluate the impact of the analog insertions on the tertiary structure of the duplexes. Nucleic acid double helices are generally categorized into three main structural forms: A-, B-, and Z-forms, with DNA/RNA hybrids largely adopting the A-form structure. CD spectra were acquired over a wavelength range of 200–350 nm, as shown in Figure 2.

The spectrum of the native duplex displayed typical A-form features, including a strong positive peak near 270 nm and a negative peak at approximately 210 nm. Comparative spectral analysis revealed minor alterations in the helical structure following chemical modification with 4′-EAE-T, 4′-BAE-T, and 4′-OAE-T. This was evident from the minor shifts in the intensities of the characteristic peaks compared to those of the unmodified duplex. Despite these changes, all modified oligonucleotides maintained a consistent spectral pattern, with the minima and maxima located at approximately 210 and 270 nm, respectively, confirming the formation of an A-type double helix with complementary RNA strands. These spectral alterations likely originated from the steric hindrance caused by the alkyl moieties attached to the *N*-ethylamino side chains of the modified oligonucleotides. Despite these slight disruptions, the overall helical conformation of the duplexes was largely unaffected.

### 2.5. Effect of Modified Duplexes on RNase Activity

**Figure 3:**
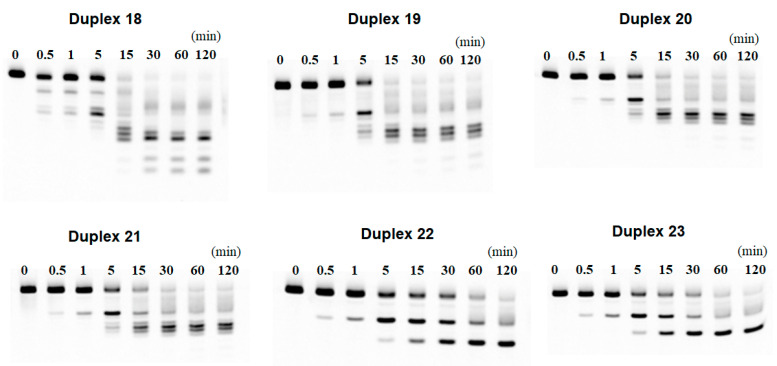
20% PAGE analysis of duplexes **18**–**23** after treatment with *E. coli* RNase H (3.5 units) at each incubation time (0, 0.25, 0.5, 1, 5, 15, 30, 60, and 120 min). Experimental conditions are described in Section 3.

Next, the RNase H-mediated degradation of the RNA strands hybridized with DNAs modified with 4′-AE-T, 4′-MAE-T, 4′-EAE-T, 4′-BAE-T, and 4′-OAE-T was systematically investigated (Table 4) (see Figure S4, Supplementary Materials). Each modified DNA sequence was paired with a complementary RNA strand labeled with fluorescein at the 5′ end. The resulting DNA/RNA hybrids were then incubated at 37 °C with Escherichia coli RNase H, and analysis was conducted at various time intervals (0, 0.25, 0.5, 1, 5, 15, 30, 60, and 120 min) using 20% denaturing polyacrylamide gel electrophoresis (PAGE). As depicted in Figure 3, duplexes **19** (containing 4′-AE-T) and **20** (4′-MAE-T) displayed RNase H cleavage activities comparable to that of the unmodified reference duplex **18**, aligning with previously reported data [27]. Similarly, duplex **21**, containing the newly synthesized 4′-EAE-T analog, also demonstrated effective RNase H activity, achieving nearly total degradation of the complementary RNA strand within 30 min. Although a slight delay in the cleavage activity was noted in the initial 15 min, no substantial differences in the overall RNase H activity were observed among the duplexes containing 4′-AE-T, 4′-MAE-T, and 4′-EAE-T analogs. In contrast, duplexes **22** (containing 4′-BAE-T) and **23** (containing 4′-OAE-T) exhibited notably lower RNase H cleavage efficiencies, whereby approximately 34% and 27% of full-length RNA remained uncleaved after 30 min of incubation, respectively. Despite this initial delay, near-complete degradation of the RNA strands was observed after 2 h. Interestingly, the degradation patterns for duplexes with 4′-AE-T, 4′-MAE-T, and 4′-EAE-T were largely similar, whereas those containing 4′-BAE-T and 4′-OAE-T produced significantly fewer cleavage fragments. The reduced efficiency of RNase H cleavage observed in the cases of 4′-BAE-T and 4′-OAE-T may be attributed to steric and/or electrostatic factors. RNase H cleavage relies on the interaction between the phosphate backbone of the DNA strand and the phosphate-binding pocket of the enzyme, which comprises basic amino acid residues such as arginine and lysine [28,29]. The 4′-aminomethyl side chains of 4′-BAE-T and 4′-OAE-T are positioned near the phosphate groups, potentially causing electrostatic repulsion with the basic amino acids of the RNase H enzyme. This repulsion, combined with insights from molecular modeling studies, suggest that the extended side chains of these analogs may hinder RNase H binding to the minor groove, thereby reducing the number of accessible cleavage sites.

**Table 4:** Sequence of DNA/RNA duplexes used in RNase H-dependent RNA hydrolysis assay.

Abbreviation of Duplex	Abbreviation of DNA and RNA	Sequence ^a^
Duplex **18**	DNA **1**	5′-d(TCTTTCTCTTTCCCTT)-3′
	RNA **2**	3′-r(AGAAAGAGAAAGGGAA)-**F**-5′
Duplex **19**	DNA **2**	5′-d(TCTTTCTCTTTCCCTT)-3′
	RNA **2**	3′-r(AGAAAGAGAAAGGGAA)-**F**-5′
Duplex **20**	DNA **3**	5′-d(TCTTTCTCTTTCCCTT)-3′
	RNA **2**	3′-r(AGAAAGAGAAAGGGAA)-**F**-5′
Duplex **21**	DNA **4**	5′-d(TCTTTCTCTTTCCCTT)-3′
	RNA **2**	3′-r(AGAAAGAGAAAGGGAA)-**F**-5′
Duplex **22**	DNA **5**	5′-d(TCTTTCTCTTTCCCTT)-3′
	RNA **2**	3′-r(AGAAAGAGAAAGGGAA)-**F**-5′
Duplex **23**	DNA **6**	5′-d(TCTTTCTCTTTCCCTT)-3′
	RNA **2**	3′-r(AGAAAGAGAAAGGGAA)-**F**-5′

^a^ red, blue, yellow, green, and purple denote 4′-AE-T (analog **1**), 4′-MAE-T (analog **2**), 4′-EAE-T (analog **3**), 4′-BAE-T (analog **4**) and 4′-OAE-T (analog **5**), respectively. **F** denotes fluorescein.

### 2.6. Evaluation of Nuclease Resistance

**Figure 4:**
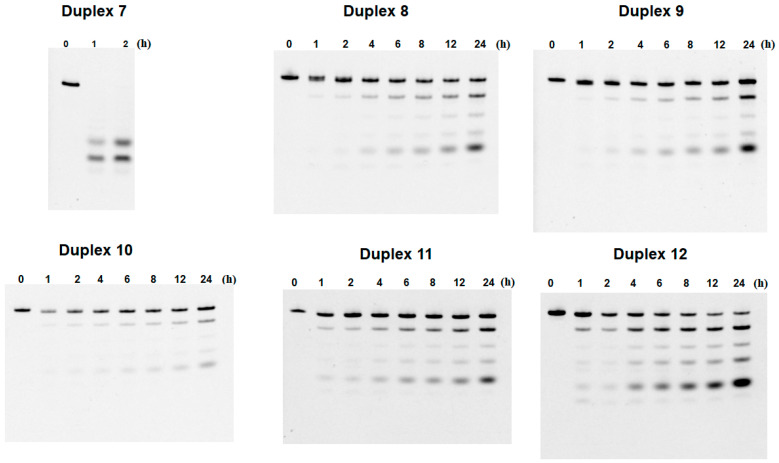
The results of nuclease resistance assay using DNAs **7**–**12**. The reaction mixtures were incubated at 37 °C. PAGE analysis of DNAs **7**–**12** at each incubation time (0, 1, 2, 4, 6, 8, 12, and 24 h) with a BS concentration of 25%.

The nuclease resistance of deoxyoligonucleotides incorporating 4′-AE-T, 4′-MAE-T, 4′-EAE-T, 4′-BAE-T, and 4′-OAE-T analogs was subsequently investigated, the sequence DNAs used in nuclease resistance assay is shown in Table 5 (see Figure S5, Supplementary Materials). Fluorescein-5′-end-labeled (300 pmol) oligonucleotides were dissolved in Opti-MEM and treated with bovine serum (BS), followed by incubation at 37 °C. Degradation was monitored at various time intervals (0, 1, 2, 4, 6, 8, 12, and 24 h) using denaturing PAGE (Figure 4). A BS concentration of 25% was employed in the assay to assess the impact of enzyme activity levels on degradation.

The results revealed distinct variations in the nuclease resistance among the various oligonucleotide analogs. Unmodified DNA (DNA **7**) exhibited rapid degradation, with a complete loss of the full-length strand within 1 h, indicating high vulnerability to nuclease activity. In line with previous reports, the inclusion of 4′-MAE-T in DNA **9** resulted in greater nuclease resistance than that of DNA **8**, which contained the simpler 4′-AE-T modification. Notably, DNA **10**, modified with the 4′-EAE-T analog, exhibited the highest resistance to nuclease degradation. This enhanced stability is likely attributable to the bulkier substituent in 4′-EAE-T, which may effectively hinder nuclease binding and activity. Remarkably, the degradation resistance of DNA **11**, containing the 4′-BAE-T modification, was similar to that of DNA **9** (4′-MAE-T), suggesting that chain extension alone does not necessarily confer further increases in stability.

Interestingly, the oligonucleotide containing 4′-OAE-T (DNA **12**), featuring the longest side chain, displayed the lowest nuclease resistance, contrary to our initial expectations. The observed decrease in resistance may be the result of steric hindrance, which possibly prevents the positively charged amino group from interacting effectively with the active site of the nuclease [30]. Furthermore, the increased lipophilicity of this analog may have facilitated interactions with the hydrophobic residues of the nuclease, thereby promoting complex formation and susceptibility to degradation. After 24 h of incubation, the percentage of remaining full-length oligonucleotides was as follows: 28.0% for DNA **8** (4′-AE-T), 34.3% for DNA **9** (4′-MAE-T), 57.0% for DNA **10** (4′-EAE-T), 36.0% for DNA **11** (4′-BAE-T), and only 10.2% for DNA **12** (4′-OAE-T); Therefore, nuclease resistance ranked as follows: 4′-EAE-T was the highest, followed by 4′-BAE-T, 4′-MAE-T, 4′-AE-T, and 4′-OAE-T.

These findings highlight the effects of side chain length and structural features on oligonucleotide stability during enzymatic degradation. The superior performance of the 4′-EAE-T analog underscores its potential as a robust modification for increasing the nuclease stability of therapeutic ASOs, while the unexpected outcome for 4′-OAE-T suggests a complex interplay between steric and hydrophobic factors that warrants further examination.

**Table 5:** Sequence of DNAs used in nuclease resistance assay.

DNA Abbreviation	Sequence ^a^
DNA **7**	5′-**F**-d(TCTTTCTCTTTCCCTT)-3′
DNA **8**	5′-**F**-d(TCTTTCTCTTTCCCTT)-3′
DNA **9**	5′-**F**-d(TCTTTCTCTTTCCCTT)-3′
DNA **10**	5′-**F**-d(TCTTTCTCTTTCCCTT)-3′
DNA **11**	5′-**F**-d(TCTTTCTCTTTCCCTT)-3′
DNA **12**	5′-**F**-d(TCTTTCTCTTTCCCTT)-3′

^a^ red, blue, yellow, green, and purple denote 4′-AE-T (analog **1**), 4′-MAE-T (analog **2**), 4′-EAE-T (analog **3**), 4′-BAE-T (analog **4**) and 4′-OAE-T (analog **5**), respectively. **F** denotes fluorescein.

### 2.7. Molecular Modeling

**Figure 5:**
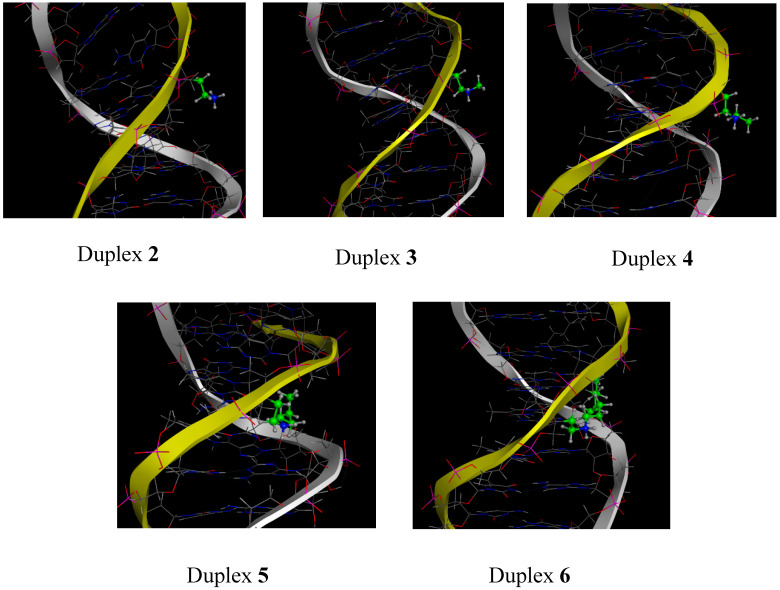
The results of modeling simulations of Duplexes **2**, **3**, **4**, **5**, and **6** containing 4′-AE-T, 4′-MAE-T, EAE-T, BAE-T, and 4′-OAE-T analogs, respectively. Simulations were performed using the MOE with the following conditions: Forcefield—Amber 14: EHT, Solvent—0.1 M NaCl. The images were captured at 5000/10,000 ps. In these images, the yellow and grey ribbons represent the DNA and RNA strands, respectively, while the green and blue balls denote the various 4′-aminoethyl side chains.

The introduction of the 4′ side chains altered RNase H cleavage patterns and affected the formation of stable double-stranded structures. To investigate the effects of the side chain on the overall three-dimensional conformation, molecular dynamics simulations were performed using the Molecular Operating Environment (MOE 2020, MOLISIS Inc., Tokyo, Japan) software. The DNA/RNA duplexes detailed in Table 2 were used as models for simulation studies, and the results are shown in Figure 5.

The simulation results indicated that in all duplexes, the 4′ side chains were predominantly arranged on the minor groove side of the DNA/RNA duplex. Notably, the side chains of 4′-AE, 4′-MAE, and 4′-EAE protruded outward from the duplex, whereas those of 4′-BAE and 4′-OAE were oriented along the minor groove within the duplex. Considering that RNase H typically approaches from the minor groove side, the alteration of its cleavage pattern likely originated from specific side chain arrangements.

Additionally, the simulation results revealed that the side chains, and in particular the carbon chain and amino group, interacted with neighboring bases and phosphate groups via hydrogen bonding. This interaction caused structural distortions, which were likely responsible for the observed decrease in the melting temperatures (*T*_m_). These results suggest that the introduction of the 4′ side chains gave rise to conformational changes that influenced both the stability of the DNA/RNA duplex and its recognition by RNase H.

### 2.8. Evaluation of Gene Expression Suppression Activity

In this study, gapmer-type ASOs were developed to specifically target the KRAS gene, one of the three main genes of the RAS gene family, comprising KRAS, HRAS, and NRAS. The dysregulated expression of any of these genes is associated with the overactivation of the RAS protein, a well-known driver of oncogenic transformation and cancer development [31]. Notably, elevated levels of RAS protein have been consistently observed in various malignant cells, emphasizing the potential therapeutic value of developing nucleic acid-based drugs targeted against RAS gene expression.

In the in vitro experiments, a cell-based assay was performed using the NCI-H460 human adenocarcinoma cell line without the aid of transfection reagents to assess the innate intracellular uptake and activity of the ASOs. The cells were plated at a density of 4000 cells/well in a 96-well plate, followed by transfection with the various ASOs, as detailed in Table 6. After incubation for 48 h, the expression level of KRAS mRNA was analyzed using reverse transcription PCR (RT-PCR). The ACTB gene (beta-actin), which is known for its stable and consistent expression, was used as a reference control, providing a baseline for data normalization and KRAS gene knockdown quantification.

**Figure 6:**
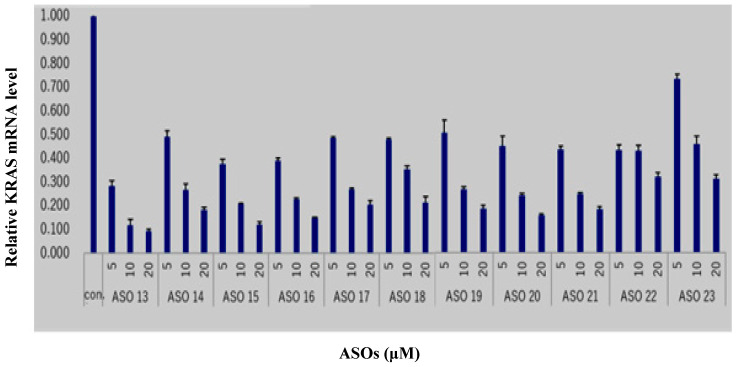
Relative KRAS mRNA levels of gapmers modified with analogs **1**, **2**, **3**, **4**, and **5** at the passenger strand were measured in NCI-H460 human adenocarcinoma cells. The cells were treated directly with gapmers (100 µM) without the use of a transfection agent. After a 24-h incubation, mRNA levels were quantified using real-time PCR. Gene expression was determined by calculating the ΔCT value relative to the ACTB gene, with the ratio calculated against the control.

The results (Figure 6) indicated that the activities of ASOs **14**–**21**, which contained AE-T, 4′-MAE-T, 4′-EAE-T, and 4′-BAE-T analogs, were similar to that of ASO **13**, which lacked these modified nucleotides. This observation indicates that 4′-EAE-T, and 4′-BAE-T can be incorporated into ASOs without compromising their anti-sense activity, suggesting the favorable potential of these novel analogs as effective alternatives to traditional PS modifications. The use of these analogs may enhance the stability and binding affinity of ASOs, while potentially reducing the off-target effects and toxicity associated with PS modifications, which are known for their non-specific interactions.

Conversely, ASO **22**, containing the 4′-OAE-T modification, demonstrated a pronounced decrease in activity. The reduced efficacy of ASO **22** is likely attributable to the significant decrease in the melting temperature (*T*_m_) of the hybrid duplex, which leads to reduced hybridization stability. Additionally, the reduced activity may have resulted from diminished RNase H recruitment and degradation activity, which are crucial for the effective cleavage of the target mRNA. These results emphasize the impact of structural modifications on the functional properties of ASOs.

**Table 6:** Sequence of oligonucleotides used in gene knockdown evaluation.

No. of ASO	Sequence (5′→3′) ^a^
ASO **13**	G^C^T^d(A^T^T^A^G^G^A^G^T^C) ^T^T^T
ASO **14**	G^C^T^d(A^T^T^A^G^G^A^G^T^C)^T^T^T
ASO **15**	G^C^T^d(A^T^T^A^G^G^A^G^T^C)^T^T^T
ASO **16**	G^C^T^d(A^T^T^A^G^G^A^G^T^C)^T^T^T
ASO **17**	G^C^T^d(A^T^T^A^G^G^A^G^T^C)^T^T^T
ASO **18**	G^C^T^d(A^T^T^A^G^G^A^G^T^C)^T^T^T
ASO **19**	G^C^T^d(A^T^T^A^G^G^A^G^T^C)^T^T^T
ASO **20**	G^C^T^d(A^T^T^A^G^G^A^G^T^C)^T^T^T
ASO **21**	G^C^T^d(A^T^T^A^G^G^A^G^T^C)^T^T^T
ASO **22**	G^C^T^d(A^T^T^A^G^G^A^G^T^C)^T^T^T
ASO **23**	G^C^T^d(A^T^T^A^G^G^A^G^T^C)^T^T^T

^a^ red, blue, yellow, green, and purple denote 4′-AE-T (analog **1**), 4′-MAE-T (analog **2**), 4′-EAE-T (analog **3**), 4′-BAE-T (analog **4**) and 4′-OAE-T (analog **5**), respectively. ^ and underline denote phosphorothioate (PS) linkage and locked nucleic acids (LNA), respectively.

## 3. Experimental Section

General remark. All chemicals and dry solvents (THF, CH_2_Cl_2_, and pyridine) were obtained from commercial sources (Fujifilm Wako, Osaka, Japan and TCI, Tokyo, Japan) and used without any further purification. Thin layer chromatography (TLC) was performed on silica gel plates precoated with fluorescent indicator visualization by UV light or by dipping into a solution of 5% (*v*/*v*) concentrated H_2_SO_4_ in a mixture of *p*-anisaldehyde and MeOH, then heating. Silica gel (63–210 mesh), obtained from Kanto Chemical Company, Tokyo, Japan, was used for column chromatography. ^1^H NMR (400, 500 MHz), ^13^C {^1^H} NMR (101 MHz), ^31^P NMR (162 MHz) were recorded on a 400, 500 MHz JEOL spectrometer. CDCl_3_ or DMSO-*d*_6_ was used as a solvent for obtaining NMR spectra. Chemical shifts (δ) are given in parts per million (ppm) from CDCl_3_ (7.26 ppm) or DMSO-*d*_6_ (2.50 ppm) for ^1^H NMR spectra; CDCl_3_ (77.2 ppm) for ^13^C NMR spectra; 80% H_3_PO_4_ (0.0 ppm) for ^31^P NMR. The abbreviations s, d, t, q and m signify singlet, doublet, triplet, quadruplet and multiplet, respectively. High resolution mass spectra (**HRMS**) were obtained in positive ion electrospray ionization (ESI-TOF).

*4′-C-[(N-Ethyl)-trifluoroacetylaminoethyl]thymidine* (**19a**). Under argon atmosphere, NaH (0.071 g, 1.79 mmol) was added to a solution of compound **17** (1.02 g, 1.49 mmol) in DMF (14.0 mL) under cooling in an ice bath, and the mixture was allowed to stir for 30 min. Then, ethyl iodide (0.36 mL, 4.47 mmol) was added to the reaction mixture and stirring was continued for a further 3 h. The mixture was extracted with H_2_O and EtOAc. The organic layer was washed with saturated NaHCO_3_ aqueous solution and brine, then dried over Na_2_SO_4_ and the solvent was removed under reduced pressure to obtain crude product **18a**, which was used in the subsequent step without further purification. Next, a solution of crude compound **18a** in CH_2_Cl_2_ (29.0 mL) was cooled for 20 min at –78 °C, and 1 M BCl_3_ (14.9 mL, 14.9 mmol) was added to the solution under argon atmosphere. After being stirred at −78 °C for 3 h, the reaction mixture was warmed to −30 °C and stirred for an additional 2 h. A mixed solution of MeOH and 28% NH_3_ aqueous solution (*v*/*v* = 4:1) was added until pH 7.0, and the mixture was filtered and concentrated under reduced pressure. The residue was purified by chromatography on silica gel (CHCl_3_/MeOH = 15:1) to give compound **19a** as a white foamy solid (0.500 g, 1.22 mmol, 82%). ^1^H NMR (400 MHz, DMSO-*d*_6_) δ: 11.29 (s, 1H), 7.70 (dd, *J* = 11.9, 0.9 Hz, 1H), 6.18–6.13 (m, 1H), 5.34 (dd, *J* = 27.2, 4.8 Hz, 1H), 5.23–5.19 (m, 1H), 4.32–4.27 (m, 1H), 3.59–3.37 (m, 6H), 2.25–2.22 (m, 1H), 2.13–2.08 (m, 1H), 1.94–1.80 (m, 2H), 1.77 (s, 3H), 1.14 (t, *J* = 7.1 Hz, 3H). ^13^C NMR (101 MHz, DMSO-*d*_6_) δ: 163.8, 158.1, 157.7, 155.1 (dd, *J*_CF_ = 35.0, 7.2 Hz), 150.5, 136.1, 116.2 (dd, *J*_CF_ = 288.5, 53.7 Hz), 109.4, 109.3, 87.8, 87.6, 83.1, 71.5, 64.2, 64.0, 43.0, 42.4, 42.0, 30.8, 28.7, 13.9, 12.3, 11.9; HRMS (ESI-TOF) *m*/*z:* Calcd. for [M + Na]^+^ C_16_H_22_F_3_N_3_Na_1_O_6_; 432.13584, found 432.13718.

4′-*C*-α-4′-*C*-[(*N*-Butyl)-trifluoroacetylaminoethyl]thymidine (**19b**). Under argon atmosphere, NaH (0.052 g, 1.30 mmol) was added to a solution of compound **17** (0.59 g, 0.87 mmol) in DMF (14.0 mL) under cooling in an ice bath, and the mixture was allowed to stir for 30 min. Then, butyl iodide (0.30 mL, 2.61 mmol) was added to the reaction mixture and stirring was continued for a further 3 h. The mixture was extracted with H_2_O and EtOAc. The organic layer was washed with saturated NaHCO_3_ aqueous solution and brine, then dried over Na_2_SO_4_ and the solvent was removed under reduced pressure to obtain crude product **18b** which was used in the subsequent step without further purification. Next, a solution of crude compound **18b** in CH_2_Cl_2_ (13.6 mL) was cooled for 20 min at −78 °C, and 1 M BCl_3_ (6.8 mL, 6.8 mmol) was added to the solution under argon atmosphere. After being stirred at −78 °C for 3 h, the reaction mixture was warmed to −30 °C and stirred for an additional 2 h. A mixed solution of MeOH and 28% NH_3_ aqueous solution (*v*/*v* = 4:1) was added until pH 7.0, and the mixture was filtered and concentrated under reduced pressure. The residue was purified by chromatography on silica gel (CHCl_3_/MeOH = 15:1) to give compound **19b** as a white foamy solid (0.245 g, 0.560 mmol, 64%). ^1^H NMR (400 MHz, DMSO-*d*_6_) δ: 11.28 (s, 1H), 7.69 (dd, *J* = 11.2, 1.1 Hz, 1H), 6.16 (td, *J* = 6.8, 4.0 Hz, 1H), 5.33 (dd, *J* = 27.5, 4.6 Hz, 1H), 5.21–5.16 (m, 1H), 4.32–4.27 (m, 1H), 3.59–3.34 (m, 6H), 2.25–2.23 (m, 1H), 2.12–2.10 (m, 1H), 1.99–1.82 (m, 2H), 1.77 (s, 3H), 1.60–1.47 (m, 2H), 1.32–1.22 (m, 2H), 0.89 (t, *J* = 6.6 Hz, 3H). ^13^C NMR (101 MHz, DMSO-*d*_6_) δ: 163.7, 155.2 (dd, *J*_CF_ = 34.5, 20.1 Hz), 150.5, 137.7, 136.1, 116.4 (d, *J*_CF_ = 288.5 Hz), 109.4, 109.3, 87.8, 87.6, 83.1, 71.6, 71.5, 64.3, 64.1, 46.9, 46.1, 43.2, 42.9, 30.8, 30.2, 28.6, 28.4, 19.5, 19.2, 13.6, 13.5, 12.3, 11.8; HRMS (ESI-TOF) *m*/*z*: Calcd. for [M + Na]^+^ C_18_H_26_F_3_N_3_Na_1_O_6_; 460.16714, found 460.16762.

*4′-C-[(N-Octyl)-trifluoroacetylaminoethyl]thymidine* (**19c**). Under argon atmosphere, NaH (0.130 g, 3.28 mmol) was added to a solution of compound **17** (1.790 g, 2.63 mmol) in DMF (26.0 mL) under cooling in an ice bath, and the mixture was allowed to stir for 30 min. Then, octyl iodide (1.42 mL, 7.88 mmol) was added to the reaction mixture and stirring was continued for a further 3 h. The mixture was extracted with H_2_O and EtOAc. The organic layer was washed with saturated NaHCO_3_ aqueous solution and brine, then dried over Na_2_SO_4_ and the solvent was removed under reduced pressure to obtain crude product **18c** which was used in the subsequent step without further purification. Next, a solution of crude compound **18c** in CH_2_Cl_2_ (35.0 mL) was cooled for 20 min at −78 °C, and 1 M BCl_3_ (21.0 mL, 21.0 mmol) was added to the solution under argon atmosphere. After being stirred at −78 °C for 3 h, the reaction mixture was warmed to −30 °C and stirred for an additional 2 h. A mixed solution of MeOH and 28% NH_3_ aqueous solution (*v*/*v* = 4:1) was added until pH 7.0, and the mixture was filtered and concentrated under reduced pressure. The residue was purified by chromatography on silica gel (CHCl_3_/MeOH = 15:1) to give compound **19c** as a white foamy solid (0.563 g, 1.14 mmol, 43%). ^1^H NMR (400 MHz, DMSO-*d*_6_) δ: 11.28 (s, 1H), 7.70 (d, *J* = 11.9 Hz, 1H), 6.18–6.14 (m, 1H), 5.32 (dd, *J* = 27.5, 4.6 Hz, 1H), 5.21–5.17 (m, 1H), 4.28 (d, *J* = 4.6 Hz, 1H), 3.56–3.39 (m, 6H), 2.25–2.20 (m, 1H), 2.12–2.09 (m, 1H), 1.92–1.81 (m, 2H), 1.75 (d, *J* = 16.0 Hz, 3H), 1.58–1.51 (m, 2H), 1.25–1.23 (m, 10H), 0.85 (t, *J* = 6.6 Hz, 3H). ^13^C NMR (101 MHz, DMSO-*d*_6_) δ: 163.8, 155.3 (dd, *J*_CF_ = 34.5, 19.2 Hz), 150.5, 136.1, 116.5 (d, *J*_CF_ = 287.5 Hz), 109.4, 109.3, 87.9, 87.7, 83.2, 71.7, 71.5, 64.3, 64.1, 47.1, 46.3, 43.2, 42.9, 31.2, 30.8, 28.6, 28.0, 26.2, 26.0, 22.1, 14.0, 12.3; HRMS (ESI-TOF) *m*/*z:* Calcd. for [M + Na]^+^ C_22_H_34_F_3_N_3_Na_1_O_6_; 516.22798, found 516.22798.

*4′-O-(4,4′-Dimethoxytrityl)-4′-C-[(N-Ethyl)-trifluoroacetylaminoethyl]thymdine* (**20a**). DMTrCl (1.25 g, 3.70 mmol) and DMAP (0.045 g, 0.370 mmol) were added to a solution of compound **19a** (0.757 g, 1.85 mmol) in pyridine (18.5 mL) under argon atmosphere, and stirred for 23 h at room temperature. The mixture was extracted with H_2_O and EtOAc. The organic layer was washed with saturated NaHCO_3_ aqueous solution and brine, dried over Na_2_SO_4_ and concentrated. The residue was purified by chromatography on silica gel (hexane/EtOAc = 1:2) to afford compound **20a** as a yellow foamy solid (0.899 g, 1.26 mmol, 68%). ^1^H NMR (400 MHz, CDCl_3_) δ: 8.12 (s, 1H), 7.42–7.27 (m, 10H), 6.85 (dd, *J* = 8.9, 2.5 Hz, 4H), 6.35–6.32 (m, 1H), 4.56–4.49 (m, 1H), 3.80 (s, 6H), 3.43–3.19 (m, 6H), 3.07 (d, *J* = 3.7 Hz, 1H), 2.42–2.37 (m, 2H), 2.04–1.90 (m, 2H), 1.55 (s, 3H), 1.17 (t, *J* = 7.1 Hz, 3H). ^13^C NMR (101 MHz, CDCl_3_) δ: 164.3, 164.2, 158.7, 157.2, 116.3 (q, *J*_CF_ = 35.1 Hz), 150.8, 150.7, 144.2, 135.9, 135.7, 135.2, 135.1, 130.1, 128.1, 128.0, 127.2, 116.5 (d, *J*_CF_ = 287.5 Hz), 113.3, 111.3, 111.1, 87.8, 87.5, 87.2, 84.5, 77.4, 73.9, 73.8, 66.8, 55.2, 43.1, 42.9, 42.2, 40.7, 40.5, 31.6, 29.7, 14.1, 12.1, 12.0, 11.9; HRMS (ESI-TOF) *m*/*z:* Calcd. for [M + Na]^+^ C_37_H_40_F_3_N_3_Na_1_O_8_; 734.26652, found 734.26602.

*4′-O-(4,4′-Dimethoxytrityl)-4′-C-[(N-Butyl)-trifluoroacetylaminoethyl]thymidine* (**20b**). DMTrCl (0.38 g, 1.12 mmol) and DMAP (0.014 g, 0.112 mmol) were added to a solution of compound **19b** (0.245g, 0.560 mmol) in pyridine (4.9 mL) under argon atmosphere, and stirred for 23 h at room temperature. The mixture was extracted with H_2_O and EtOAc. The organic layer was washed with saturated NaHCO_3_ aqueous solution and brine, dried over Na_2_SO_4_ and concentrated. The residue was purified by chromatography on silica gel (hexane/EtOAc = 1:2) to afford compound **20b** as a yellow foamy solid (0.317 g, 0.429 mmol, 77%). ^1^H NMR (400 MHz, CDCl_3_) δ: 8.60 (s, 1H), 7.42–7.26 (m, 10H), 6.86–6.83 (m, 4H), 6.36–6.30 (m, 1H), 4.50 (s, 1H), 3.79 (s, 6H), 3.54–3.24 (m, 6H), 3.19 (d, *J* = 9.6 Hz, 1H), 2.46–2.33 (m, 2H), 2.08–1.86 (m, 2H), 1.55 (s, 3H), 1.51 (q, *J* = 8.1 Hz, 2H), 1.33–1.22 (m, 2H), 0.91 (t, *J* = 7.1 Hz, 3H). ^13^C NMR (101 MHz, CDCl_3_) δ: 164.3, 164.2, 158.8, 157.0, 156.7 (dd, *J*_CF_ = 35.9, 25.4 Hz), 150.8, 150.7, 144.2, 135.8, 135.7, 135.2, 135.1, 130.1, 128.1, 128.0, 127.2, 118.0, 116.5 (d, *J*_CF_ = 287.5 Hz), 113.3, 111.3, 111.1, 87.7, 87.5, 87.3, 87.2, 84.6, 77.4, 73.9, 73.8, 66.9, 55.2, 48.1, 46.8, 43.7, 43.4, 40.7, 40.4, 31.6, 30.8, 29.7, 28.9, 20.0, 19.7, 13.7, 13.6, 12.0; HRMS (ESI-TOF) *m*/*z*: Calcd. for [M + Na]^+^ C_39_H_44_F_3_N_3_Na_1_O_8_; 762.29782, found 762.29484.

*4′-O-(4,4′-Dimethoxytrityl)-4′-C-[(N-Octyl)-trifluoroacetylaminoethyl]thymidine* (**20c**). DMTrCl (0.579 g, 1.71 mmol) and DMAP (0.027 g, 0.228 mmol) were added to a solution of compound **19c** (0.563g, 1.14 mmol) in pyridine (12.0 mL) under argon atmosphere, and stirred for 23 h at room temperature. The mixture was extracted with H_2_O and EtOAc. The organic layer was washed with saturated NaHCO_3_ aqueous solution and brine, dried over Na_2_SO_4_ and concentrated. The residue was purified by chromatography on silica gel (hexane/EtOAc = 1:2) to afford compound **20c** as a yellow foamy solid (0.654 g, 0.822 mmol, 72%). ^1^H NMR (400 MHz, CDCl_3_) δ: 8.07 (s, 1H), 7.43–7.27 (m, 10H), 6.86–6.83 (m, 4H), 6.36–6.27 (m, 1H), 4.53–4.49 (m, 1H), 3.80 (s, 6H), 3.40–3.20 (m, 6H), 3.18–3.15 (m, 1H), 2.42–2.35 (m, 2H), 2.07–1.89 (m, 3H), 1.57 (s, 3H), 1.55 (d, *J* = 0.9 Hz, 1H), 1.30–1.25 (m, 10H), 0.89 (t, *J* = 7.1 Hz, 3H). ^13^C NMR (101 MHz, CDCl_3_) δ: 164.1, 164.0, 158.9, 157.0 (q, *J*_CF_ = 36.7 Hz), 144.2, 135.8, 135.6, 135.3, 135.2, 135.1, 130.2, 128.2, 127.3, 116.5 (d, *J*_CF_ = 287.5 Hz), 113.4, 111.4, 111.2, 87.7, 87.4, 87.3, 84.7, 84.6, 77.4, 74.1, 74.0, 67.0, 66.9, 47.2, 43.8, 43.4, 40.8, 40.4, 31.9, 31.8, 29.8, 29.4, 29.2, 29.0, 26.9, 26.8, 26.6, 22.7, 14.2, 12.2, 12.1; HRMS (ESI-TOF) *m*/*z:* Calcd. for [M + Na]^+^ C_43_H_52_F_3_N_3_Na_1_O; 818.36042, found 818.35808.

*1-[3′-O-(2-Cyanoethyl-N, N-diisopropylphosphoramidite)-4′-O-(4,4′-Dimethoxytrityl)-4′-C-[(N-Ethyl)-trifluoroacetylaminoethyl]thymidine* (**21a**). *N,N*-Disopropylethylamine (DIPEA, 0.790 mL, 4.54 mmol) and *N,N*-disoproylchlorophosphoramidite (CEP-Cl, 0.405 mL, 1.82 mmol) were added to a solution of compound 20a (0.6465 g, 0.908 mmol) in THF (6.5 mL) under argon atmosphere, and stirred for 1.5 h at room temperature. The mixture was extracted with saturated NaHCO_3_ aqueous solution and CHCl_3_. The organic layer was washed with saturated NaHCO_3_ aqueous solution and brine, dried over Na_2_SO_4_ and concentrated. The residue was purified by chromatography on silica gel (hexane/EtOAc = 3:2) to afford compound 21a as a white foamy solid (0.684 g, 0.75 mmol, 82%). ^31^P NMR (162 MHz, CDCl_3_) δ: 150.6, 150.3, 150.2, 150.1; HRMS (ESI-TOF) *m*/*z:* Calcd. for [M + Na]^+^ C_46_H_57_F_3_N_5_Na_1_O_9_P_1_; 934.37437, found 934.37521.

*1-[3′-O-(2-Cyanoethyl-N, N-diisopropylphosphoramidite)-4′-O-(4,4′-Dimethoxytrityl)-4′-C-[(N-butyl)-trifluoroacetylaminoethyl]thymidine* (**21b**). *N,N*-Disopropylethylamine (DIPEA, 0.64 mL, 3.64 mmol) and *N,N*-disoproylchlorophosphoramidite (CEP-Cl, 0.365 mL, 1.46 mmol) were added to a solution of compound 20b (0.317 g, 0.429 mmol) in THF (5.4 mL) under argon atmosphere, and stirred for 1.5 h at room temperature. The mixture was extracted with saturated NaHCO_3_ aqueous solution and CHCl_3_. The organic layer was washed with saturated NaHCO_3_ aqueous solution and brine, dried over Na_2_SO_4_ and concentrated. The residue was purified by chromatography on silica gel (hexane/EtOAc = 3:2) to afford compound 21b as a white foamy solid (0.522 g, 0.555 mmol, 76%) ^31^P NMR (162 MHz, CDCl_3_) δ: 150.5, 150.3, 150.1, 150.1; HRMS (ESI-TOF) *m*/*z:* Calcd. for [M + Na]^+^ C_48_H_61_F_3_N_5_Na_1_O_9_P_1_; 962.40567, found 962.40123.

*1-[3′-O-(2-Cyanoethyl-N, N-diisopropylphosphoramidite)-4′-O-(4,4′-Dimethoxytrityl)-4′-C-[(N-octyl)-trifluoroacetylaminoethyl]thymidine* (**21c**). *N,N*-Disopropylethylamine (DIPEA, 0.72 mL, 4.11 mmol) and *N,N*-disoproylchlorophosphoramidite (CEP-Cl, 0.37 mL, 1.64 mmol) were added to a solution of compound 20c (0.654 g, 0.82 mmol) in THF (6.5 mL) under argon atmosphere, and stirred for 1.5 h at room temperature. The mixture was extracted with saturated NaHCO_3_ aqueous solution and CHCl_3_. The organic layer was washed with saturated NaHCO_3_ aqueous solution and brine, dried over Na_2_SO_4_ and concentrated. The residue was purified by chromatography on silica gel (hexane/EtOAc = 3:2) to afford compound 21c as a white foamy solid (0.725 g, 0.73 mmol, 88%). ^31^P NMR (162 MHz, CDCl_3_) δ: 150.5, 150.3, 150.1, 150.1; HRMS (ESI-TOF) *m*/*z:* Calcd. for [M + Na]^+^ C_52_H_69_F_3_N_5_Na_1_O_9_P_1_; 1018.46827, found 1018.46510.

Solid-Phase Oligonucleotide Synthesis: Oligonucleotide synthesis was conducted on a DNA/RNA synthesizer employing the phosphoramidite methodology. The DNA phosphoramidites and LNA phosphoramidites were prepared in 0.1 M acetonitrile (MeCN), except for LNA-C, which was dissolved in a 1:1 (*v*/*v*) mixture of dichloromethane (CH_2_Cl_2_) and MeCN. For 4′-AE-T and 4′-MAE-T phosphoramidites, a 0.15 M solution in MeCN was used. Sulfurization was performed utilizing 0.2 M solution of xanthan hydride in pyridine. Upon completion of the synthesis, DNA oligomers were detached from the CPG beads and their acyl groups deprotected by incubation in ammonia (NH₃) solution at 55 °C for 12 h. When oligomers included 4′-AE-T, 4′-MAE-T, 4′-EAE-T, 4′-BAE-T or 4′-OAE-T units, cyanoethyl groups were removed selectively before the ammonia treatment by exposing the oligomers to 10% diethylamine in MeCN for 5 min. For RNA synthesis, oligomers were cleaved from the CPG beads and deprotected using a mixture of concentrated NH_3_ and 40% methylamine in a 1:1 (*v*/*v*) ratio at 65 °C for 10 min. Subsequently, the 2′-O-TBDMS protecting groups were eliminated by treating the oligomers with triethylamine trihydrofluoride (Et_3_N·3HF) in dimethyl sulfoxide (DMSO) at 65 °C for 90 min. The reaction was stopped by adding a 0.1 M triethylammonium acetate (TEAA) buffer at pH 7.0, followed by desalting through a Sep-Pak C18 cartridge. The synthesized oligonucleotides were purified by 20% PAGE under denaturing conditions in the presence of 7 M urea. Finally, the molecular weights of the purified DNA and RNA oligomers were verified using MALDI-TOF mass spectrometry.

MALDI-TOF/MS analysis of oligonucleotides: The spectra were obtained with a time-of-light mass spectrometer equipped with a nitrogen laser (337 nm, 3 ns pulse). A solution of 3-hydroxypicolinic acid (3-HPA) and diammonium hydrogen citrate in H_2_O was used as the matrix. Data of synthetic ON: ASO **1** *m*/*z* = 4714.22 (calcd for C_154_H_203_N_38_O_104_P_15_ [M − H]^−^, 4715.06); ASO **2** *m*/*z* = 4886.58 (calcd for C_162_H_223_N_42_O_104_P_15_ [M − H]^−^, 4886.69); ASO **3** *m*/*z* = 4942.76 (calcd for C_166_H_227_N_42_O_104_P_15_ [M − H]^−^, 4943.45); ASO **4** *m*/*z* = 4998.83 (calcd for C_170_H_239_N_42_O_104_P_15_ [M − H]^−^, 4999.56); ASO **5** *m*/*z* = 5112.15 (calcd for C_178_H_255_N_42_O_104_P_15_ [M − H]^−^, 5111.76); ASO **6**: *m*/*z* = 5336.35 (calcd for C_194_H_287_N_42_O_104_P_15_ [M − H]^−^, 5336.20); ASO **7** *m*/*z* = 5253.4 (calcd for C_181_H_247_N_42_O_113_P_16_ [M − H]^−^, 5253.53); ASO **8** *m*/*z* = 5424.72 (calcd for C_215_H_281_N_46_O_127_P_16_ [M − H]^−^, 5425.83); ASO **9** *m*/*z* = 5481.95 (calcd for C_219_H_389_N_78_O_121_P_16_ [M − H]^−^, 5481.95); ASO **10** *m*/*z* = 5536.06 (calcd for C_223_H_297_N_78_O_121_P_16_ [M − H]^−^, 5538.08); ASO **11** *m*/*z* = 5650.13 (calcd for C_231_H_313_N_58_O_88_P_16_ [M − H]^−^, 5650.27); ASO **12** *m*/*z* = 5874.71 (calcd for C_247_H_313_N_62_O_88_P_16_ [M − H]^−^, 5874.68); ASO **13** *m*/*z* = 5323.99 (calcd for C_165_H_202_N_55_O_89_P_15_S_15_ [M − H]^−^, 5325.32); ASO **14** *m*/*z* = 5366.96 (calcd for C_167_H_207_N_56_O_89_P_15_S_15_ [M − H]^−^, 5368.38); ASO **15** *m*/*z* = 5367.96 (calcd for C_167_H_207_N_56_O_89_P_15_S_15_ [M − H]^−^, 5368.38); ASO **16** *m*/*z* = 5381.19 (calcd for C_168_H_209_N_55_O_89_P_15_S_15_ [M − H]^−^, 5382.41); ASO **17** *m*/*z* = 5381.27 (calcd for C_168_H_209_N_55_O_89_P_15_S_15_ [M − H]^−^, 5382.41); ASO **18** *m*/*z* = 5395.31 (calcd for C_169_H_211_N_56_O_89_P_15_S_15_ [M − H]^−^, 5396.44); ASO **19** *m/z* = 5396.56 (calcd for C_169_H_211_N_56_O_89_P_15_S_15_ [M − H]^−^, 5396.44); ASO **20** *m*/*z* = 5424.42 (calcd for C_171_H_215_N_56_O_89_P_15_S_15_ [M − H]^−^, 5424.49); ASO **21** *m*/*z* = 5423.41 (calcd for C_171_H_215_N_56_O_89_P_15_S_15_ [M − H]^−^, 5424.49); ASO **22** *m*/*z* = 5480.54 (calcd for C_175_H_223_N_56_O_89_P_15_S_15_ [M − H]^−^, 5480.51); ASO **23** *m*/*z* = 5479.61 (calcd for C_175_H_223_N_56_O_89_P_15_S_15_ [M − H]^−^, 5480.51); RNA **1** *m*/*z* = 5371.32 (calcd for C_186_H_228_N_84_O_114_P_15_ [M − H]^−^, 5072.11); RNA **2** *m*/*z* = 5420.79 (calcd for C_213_H_252_N_42_O_114_P_16_ [M − H]^−^, 5610.58); RNA **3** *m*/*z* = 5071.72 (calcd for C_179_H_224_N_67_O_120_P_15_ [M − H]^−^, 5072.11).

Thermal denaturation study: DNA (600 pmol) and RNA (600 pmol) were dissolved in a buffer of 10 mM sodium phosphate (200 µL, pH 7.0) containing 100 mM NaCl, and this solution was heated for 5 min at 100 °C. Then the solution was cooled gradually to room temperature for more than 1 h and used for the thermal denaturation study. Thermally induced transitions were monitored at 260 nm with a UV/Vis spectrometer fitted with temperature controller in quartz cuvettes with a path length of 1.0 cm. The sample temperature increased by 0.5 °C/min. The 50% melting temperature was calculated by the midline method from the sigmoid curve obtained from the measurement. The thermodynamic parameters of the duplexes on duplex formation were determined by calculations based on the slope of a 1/*T*_m_ vs. ln (C_T_/4) plot, where CT (1, 3, 6, 12, 15, 21, 16, 30, and 60 μM) is the total concentration of single strands.

CD spectroscopy: All CD spectra were measured at 25 °C. DNA (1200 pmol) and RNA (1200 pmol) were dissolved in a buffer of 10 mM sodium phosphate (pH 7.0) containing 100 mM NaCl to give final duplex concentration of 4.0 μM. The duplex hybrid was obtained through annealing by heating for 5 min at 100 °C and then gradually cooling toroom temperature over 1 h. The following instrument settings were used: resolution, 0.1 nm; response, 1.0 s; speed, 50 nm/min; accumulation, 10.

Hydrolysis of DNA/RNA duplex with Escherichia coli RNase H: DNA (300 pmol) and RNA (1500 pmol) were dissolved in a buffer of 50 mM Tris HCl (pH 8.0) and 75 mM KCl, 3 mM MgCl_2_, 10 mM dithiothreitol. The solutions were heated for 3 min at 100 °C. Following the solution was cooled gradually to room temperature over 1 h. Following, E. coli RNaseH (3.5 units) was added to the solutions and incubated at 37 °C. Aliquots (5 μL) were taken at 0, 0.25, 0.5, 1,5, 15, 30, 60 min and mixed with formamide (15 μL) on ice. Each sample was analyzed by electrophoresis in 20% PAGE at 20 mA for 2.5 h. The gel was visualized using Luminescent Image analyzer LAS-4000 (Fujifilm, Tokyo, Japan).

Evaluation of nuclease resistance: A quantity of 300 pmol of each oligonucleotide was first dried under reduced pressure and then reconstituted in 37.5 µL of Opti-MEM. An aliquot of 1.1 µL was taken as the 0-min control sample and mixed with 5 µL of loading buffer. The remaining samples were incubated at 37 °C, after which bovine serum (BS) was added at final concentrations of 25% (36.4 µL). At the designated time points, the reactions were halted by transferring 2.4 µL of the mixture into an Eppendorf tube preloaded with 10 µL of loading buffer. Subsequently, 5 µL of each sample was subjected to electrophoresis on a 20% polyacrylamide gel (PAGE) at 500 V and 20 mA for approximately 3 h. Imaging of the gel was carried out using the Luminescent Image Analyzer LAS-4000 (Fujifilm).

Molecular Modeling: Simulations were carried out by Molecular Operating Environment (MOE) underspecificconditions. Amber 14: EHT Forcefield was employed with a solvent environment of 0.1 M NaCl. The DNA strand (5′-d(TCTTTCTCTTTCCCTT)-3′) and the RNA strand (3′-r(AAGGGAAAGAGAAAGA)-5′) were constructed using Amber14: EHT forcefield and subsequently subjected to energy minimization. Then, modifications involving various 4′ side chains were introduced directly to the RNA strand to form duplexes **2** through **6**. These duplexes underwent energy minimization, protonation using default conditions, and solvent was added. Following solvation, Energy Minimization was repeated under default parameters, and Dynamics simulations; NAMD (default conditions; energy minimizing; 10 ps, heating; 100 ps, NVT equilibrating; 100 ps, NPT equilibrating; 200 ps, and production run; 10,000 ps) was conducted.

Evaluation of gene expression suppression ability: The gene expression suppression ability was evaluated without using a transfection reagent using reverse transcription PCR. NCI-H460 cells were prepared to 80,000 cells/mL, and 50 µL was placed in each well of a 96-well plate. After confirming the adhesion of the cells to the bottom, gapmer-type ASO (100 µM), dissolved in PBS buffer, was added to the final concentration. After that, the wells were allowed to stand for 1 h in a CO_2_ incubator at 37 °C, and then, 50 µL of D-MEM was added to each well and cultured in a CO_2_ incubator at 37 °C. 24 h after seeding the cells, 50 µL of D-MEM was added to each well and cultured in a CO_2_ incubator at 37 °C for 48 h. The medium was removed, and 100 µL of PBS (-) was added to each well, washed, and then removed from the wells. Next, 50 µL of cell lysis solution (Lysis solution: DNase1 = 49.6:0.4) was added to each well, and the plate was shaken for 5 min and then left to stand for 5 min. Next, 5 µL of stop solution was added to each well, and the plate was shaken for 3 min and then left to stand for 2 min to prepare cell lysate. Next, 30 µL of reverse transcription buffer (2×Fast Advanced RT buffer: 20×Fast Advanced RT Enzyme mix: H_2_O = 10:1:1) was added to the reverse transcription plate, and 20 µL of the prepared lysate was added, followed by centrifugation at 2000 rpm for 2 min. Next, cDNA was prepared by reverse transcription using a thermal cycler. (37 °C, 30 min → 95 °C, 5 min → 4 °C) 16 µL of PCR cocktail (TaqMan Fast Advanced Master Mix: Primer Probe: H_2_O = 5:2:1) was added to a real-time PCR plate, and 4 µL of cDNA template was added and centrifuged at 2000 rpm for 2 min. Then, mRNA was quantified by real-time PCR. The expression level of the gene was calculated by calculating the ΔCT value based on the ACTB gene present in the cells, and the ratio was calculated based on the control.

## 4. Conclusions

This study reports the synthesis of three novel 4′-C-aminoalkyl-modified thymidine derivatives: 4′-*C*-(*N*-ethyl)aminoethylthymidine (4′-EAE-T), 4′-*C*-(*N*-butyl)aminoethylthymidine (4′-BAE-T), and 4′-*C*-(*N*-octyl)aminoethylthymidine (4′-OAE-T). Additionally, the study evaluates the potential of these derivatives in enhancing the therapeutic properties of antisense oligonucleotides (ASOs). The RNA binding affinities of DNA strands incorporating the 4′-EAE and 4′-BAE analogs were comparable to those of the previously reported 4′-MAE analog. Conversely, the 4′-OAE analog showed a significant decline in RNA binding affinity. In addition, all modifications demonstrated remarkably high resistance to nuclease degradation. Among these modifications, the 4′-EAE analog stands out owing to its superior nuclease resistance in addition to maintaining robust RNase H activity and thermal stability (*T*_m_) similar to that of previously synthesized analogs. Molecular modeling revealed that the unique orientation of the 4′-aminoalkyl side chains within the minor groove of the ASO/RNA duplex and their hydrogen bonding interactions with adjacent phosphate groups contributed to the observed differences in duplex stability and enzymatic activity. Furthermore, in vitro cellular experiments were conducted to evaluate the suppression of KRAS expression attained by the various ASOs in NCI-H460 human adenocarcinoma cells without the use of transfection reagents. The modified gapmer-type ASOs exhibited gene suppression activities comparable to those of unmodified controls, with the exception of the 4′-OAE-containing ASO, which displayed inferior performance. These findings position the 4′-EAE analog as a valuable modification for designing gapmer-type ASOs with enhanced stability, efficacy, and reduced toxicity.

## Data Availability

The original contributions presented in this study are included in the article/Supplementary Materials. Further inquiries can be directed to the corresponding author.

## Supplementary Materials

The following supporting information can be downloaded at: https://www.mdpi.com/article/10.3390/molecules30030581/s1. UV melting profiles of duplex **1**–**6**, **7**–**13**, and **14**–**17**, cleaving profile of duplex **18**–**23** with RNase H enzyme, evaluation of nuclease resistance using DNAs **7**–**12**, and copies of NMR spectra (^1^H, ^13^C and ^31^P).
