# Supplementary material for: Synthesis and Application of 4′-C-[(N-alkyl)aminoethyl]thymidine Analogs for Optimizing Oligonucleotide Properties
Source: Molecules. 2025 Jan 27;30(3):581. doi: 10.3390/molecules30030581 (PMC11820600; doi:10.3390/molecules30030581)

Supplementary Information

Synthesis and Application of 4′-*C*-[(*N*-alkyl)aminoethyl]thymidine Analogs for Optimizing Oligonucleotide Properties

Kota Fujiki [1], Yuri Kakisawa [2], Elsayed M. Mahmoud [2,3] and Yoshihito Ueno [1,2,4,5,*]

[1] Department of Life Science and Chemistry, The Graduate School of Natural Science and Technology, Gifu University, 1-1 Yanagido, Gifu 501-1193, Japan; fujiki.kouta.t9@s.gifu-u.ac.jp

[2] Course of Applied Life Science, Faculty of Applied Biological Sciences, Gifu University, 1-1 Yanagido, Gifu 501-1193, Japan; kkrsw113@gmail.com (Y.K.); elsayed.maher.mahmoud.saleh.r8@f.gifu-u.ac.jp (E.M.M.)

[3] Department of Pharmaceutical Organic Chemistry, Faculty of Pharmacy, Zagazig University, Zagazig 44519, Egypt

[4] United Graduate School of Agricultural Science, Gifu University, 1-1 Yanagido, Gifu 501-1193, Japan

[5] Center for One Medicine Innovative Translational Research (COMIT), Tokai National Higher Education and Research System, Gifu University, 1-1 Yanagido, Gifu 501-1193, Japan

* Correspondence: ueno.yoshihito.e7@f.gifu-u.ac.jp; Tel.: +81-58-293-2919; Fax: +81-58-293-2919

Table of Contents

1. Figure S1. UV melting profiles of Duplex **1**–**6** in 10 mM phosphate / 100 mM NaCl buffer……S3

2. Figure S2. UV melting profiles of Duplex **7**–**13** in 10 mM phosphate / 1.0 M NaCl buffer. ...…...S4

3. Figure S3. UV melting profiles of Duplex **14**–**17** in 10 mM phosphate / 1.0 M NaCl buffer.….S5

4. Figure S4. Cleaving profile of Duplex **18**–**23** with RNase H enzyme.….…………………………S6

5. Figure S5. Evaluation of nuclease resistance using DNA **7**–**12**.….………………………………..S7

6. Copies of NMR spectra (^{1}H, ^{13}C and ^{31}P)........……………………………………………..S8

1. Figure S1. UV melting profiles of Duplex **1**–**6** in 10 mM phosphate / 100 mM NaCl buffer.

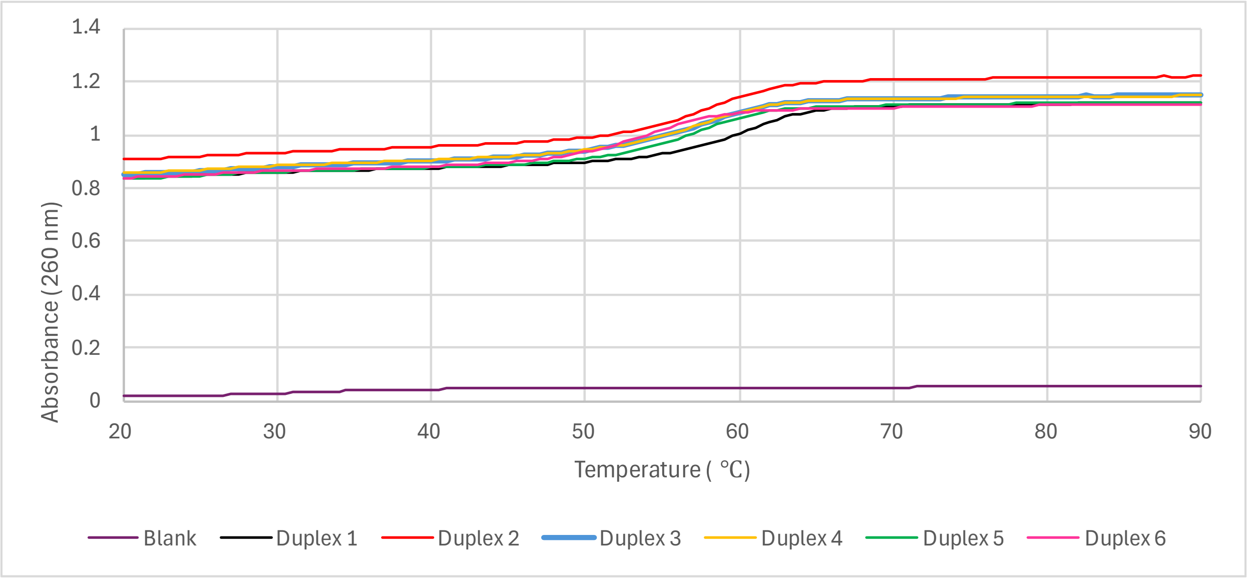

Figure S1. UV melting profiles of Duplex **1**–**6** in 10 mM phosphate / 100 mM NaCl buffer. Duplex **1** represents the unmodified control, while Duplexes **2**, **3**, **4**, **5**, and **6** correspond to duplexes incorporating the modified analogs 4'-AE-T (**1**), 4'-MAE-T (**2**), 4'-EAE-T (**3**), 4'-BAE-T (**4**) and 4'-OAE-T (**5**), respectively.

2. Figure S2. UV melting profiles of Duplex **7–13** in 10 mM phosphate / 100 mM NaCl buffer.

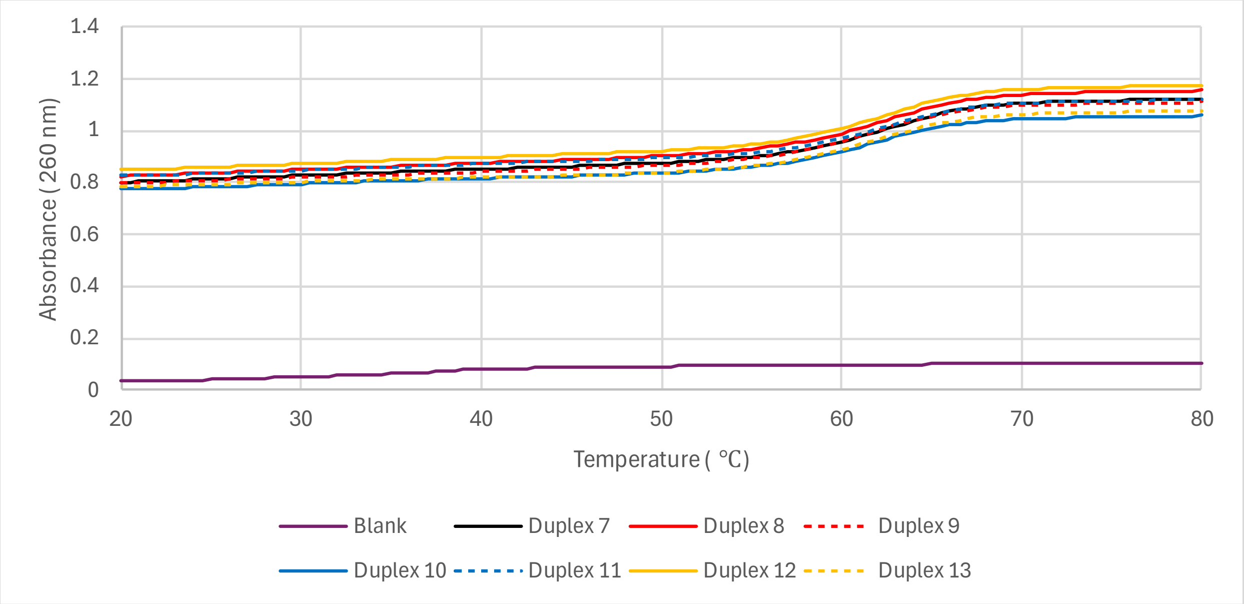

Figure S2. UV melting profiles of Duplex **7–13** in 10 mM phosphate / 100 mM NaCl buffer. Duplex **7** represents the unmodified control, while Duplexes **8**, **9**, **10**, **11**, **12** and **13** correspond to duplexes incorporating the modified analogs 4'-AE-T (**1**) near 5' end, 4'-AE-T (**1**) near 3' end, 4'-MAE-T (**2**) near 5' end, 4'-MAE-T (**2**) near 3' end, 4'-EAE-T (**3**) near 5' end, and 4'-EAE-T (**3**) near 3' end, respectively.

3. Figure S3. UV melting profiles of Duplex **14–17** in 10 mM phosphate / 100 mM NaCl buffer.

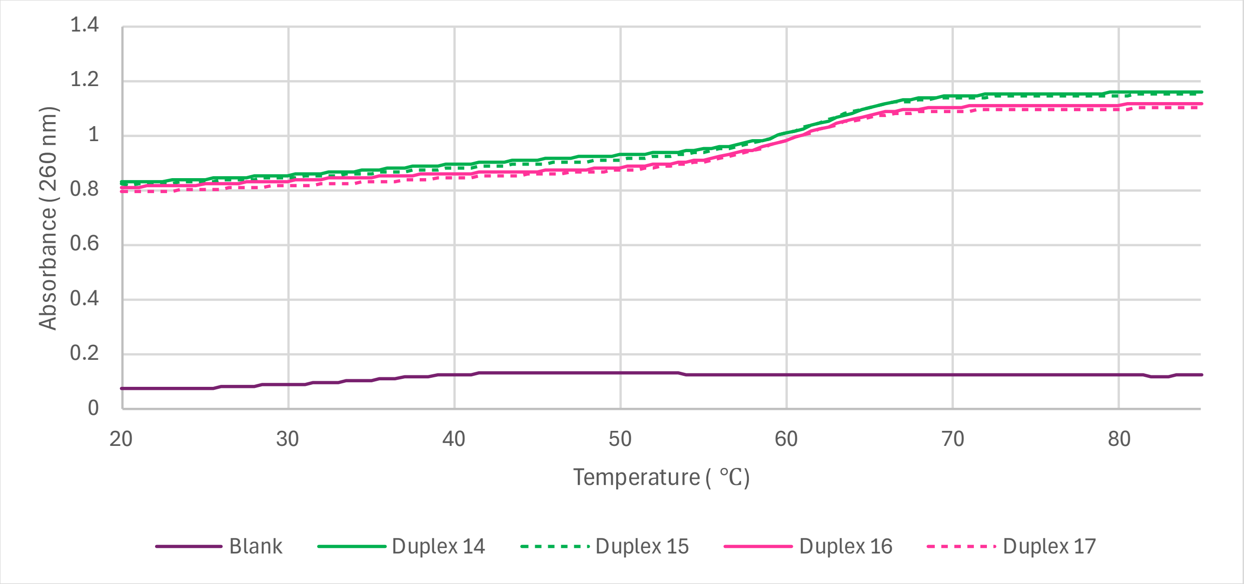

Figure S3. UV melting profiles of Duplex **14–17** in 10 mM phosphate / 100 mM NaCl buffer. Duplexes **14**, **15**, **16** and **17** correspond to duplexes incorporating the modified analogs 4'-BAE-T (**4**) near 5' end, 4'-BAE-T (**4**) near 3' end, 4'-OAE-T (**5**) near 5' end, and 4'-OAE-T (**5**) near 3' end, respectively.

4. Figure S4 Cleaving profile of Duplex **18–23** with RNase H enzyme.

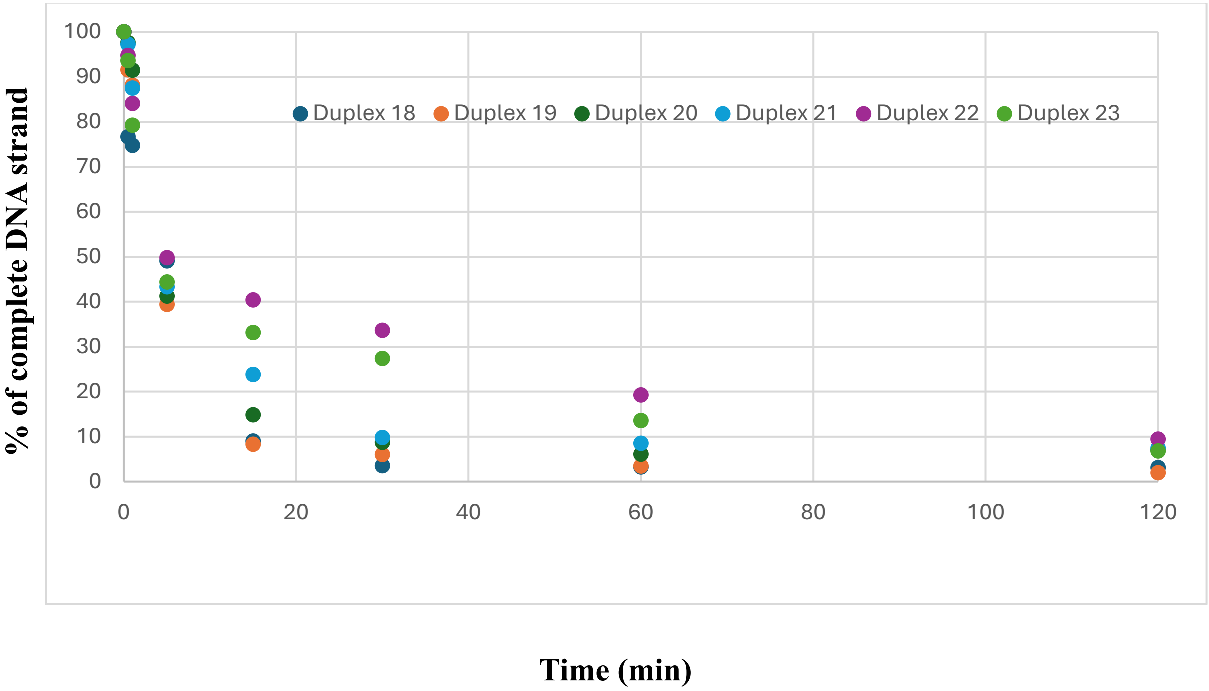

Figure S4. Cleaving profile of Duplex **18–23** with RNase H enzyme. Duplex **18** represents the unmodified control, while Duplexes **19**, **20**, **21**, **22**, and **23** correspond to duplexes incorporating the modified analogs 4'-AE-T (**1**), 4'-MAE-T (**2**), 4'-EAE-T (**3**), 4'-BAE-T (**4**) and 4'-OAE-T (**5**), respectively.

5. **Figure S5** Evaluation of nuclease resistance using DNA **7–12**

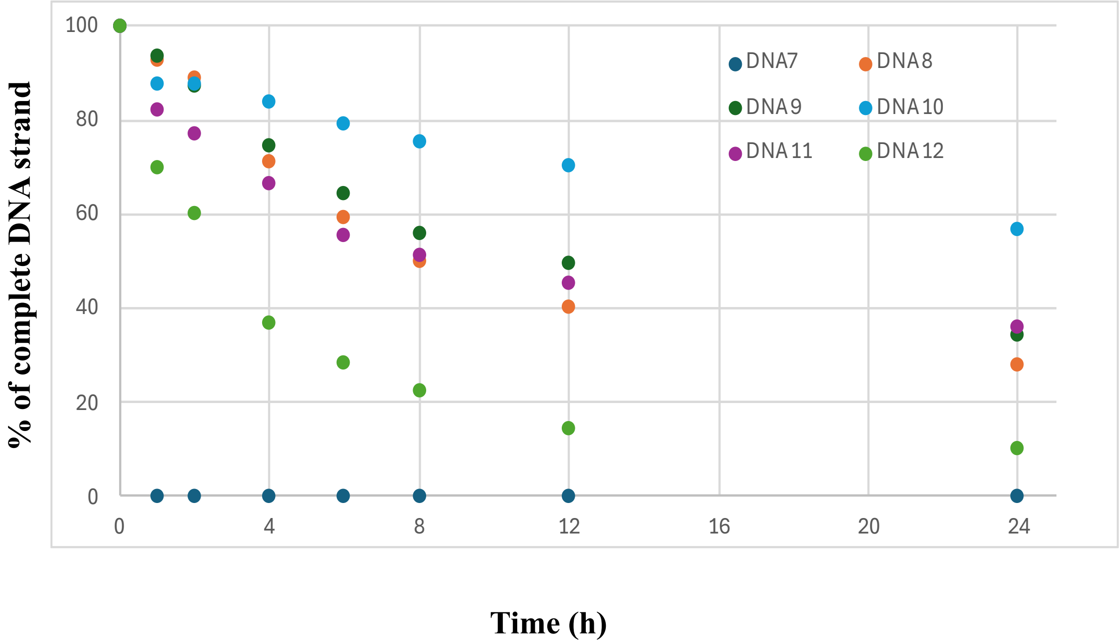

Figure S5. Evaluation of nuclease resistance using DNA **7–12**. DNA **7** represents the unmodified control, while DNAs **8**, **9**, **10**, **11**, and **12** correspond to DNAs incorporating the modified analogs 4'-AE-T (**1**), 4'-MAE-T (**2**), 4'-EAE-T (**3**), 4'-BAE-T (**4**) and 4'-OAE-T (**5**), respectively.

6. NMR spectra (^{1}H, ^{13}C and ^{31}P).

^{1}HNMR (400 MHz, DMSO-d_6) **19a**

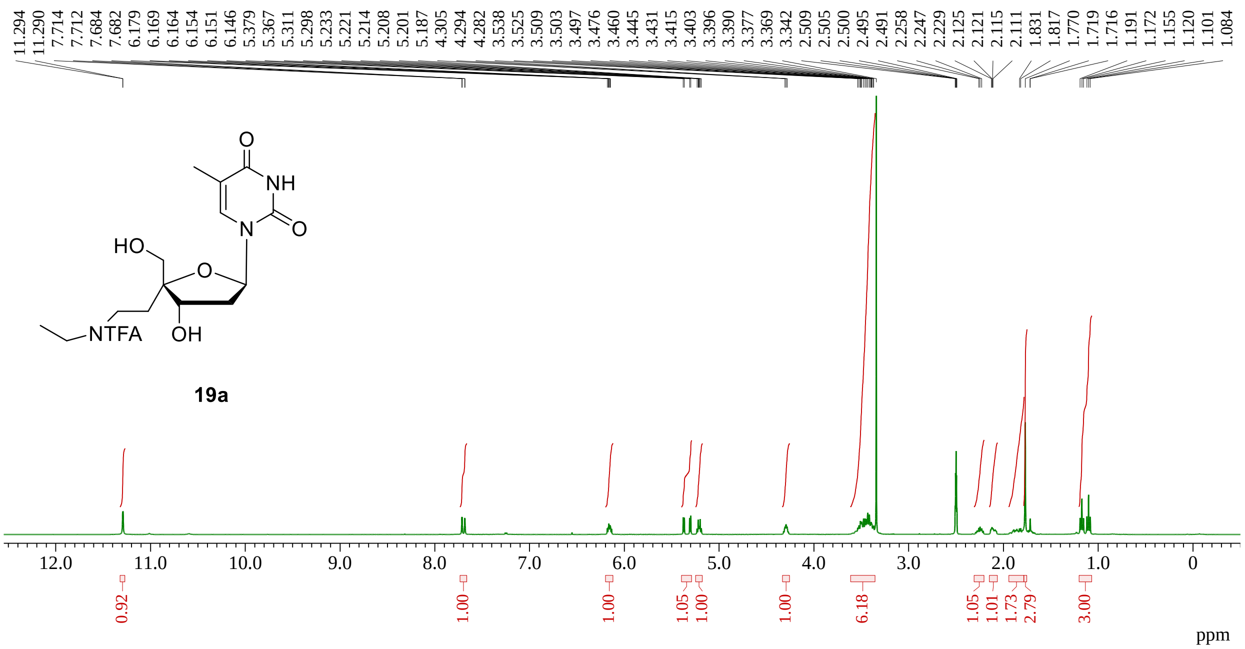

^{13}CNMR (101 MHz, DMSO-d_6) **19a**

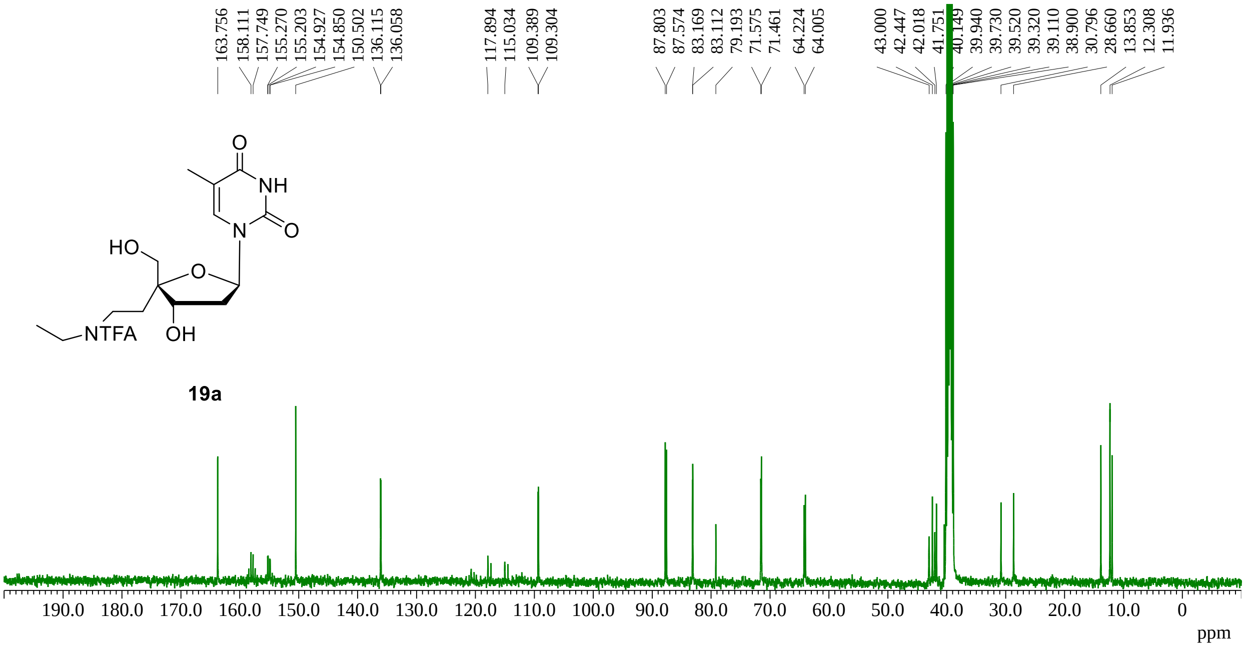

^{1}HNMR (400 MHz, DMSO-d_6) **19b**

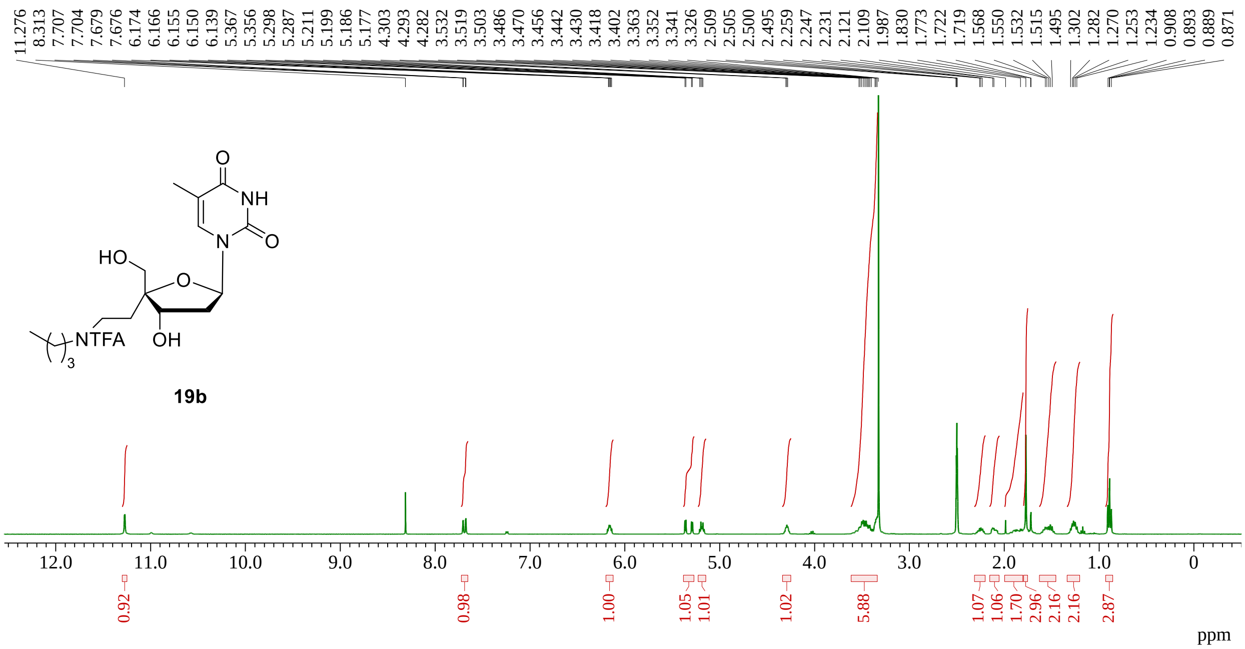

^{13}CNMR (101 MHz, DMSO-d_6) **19b**

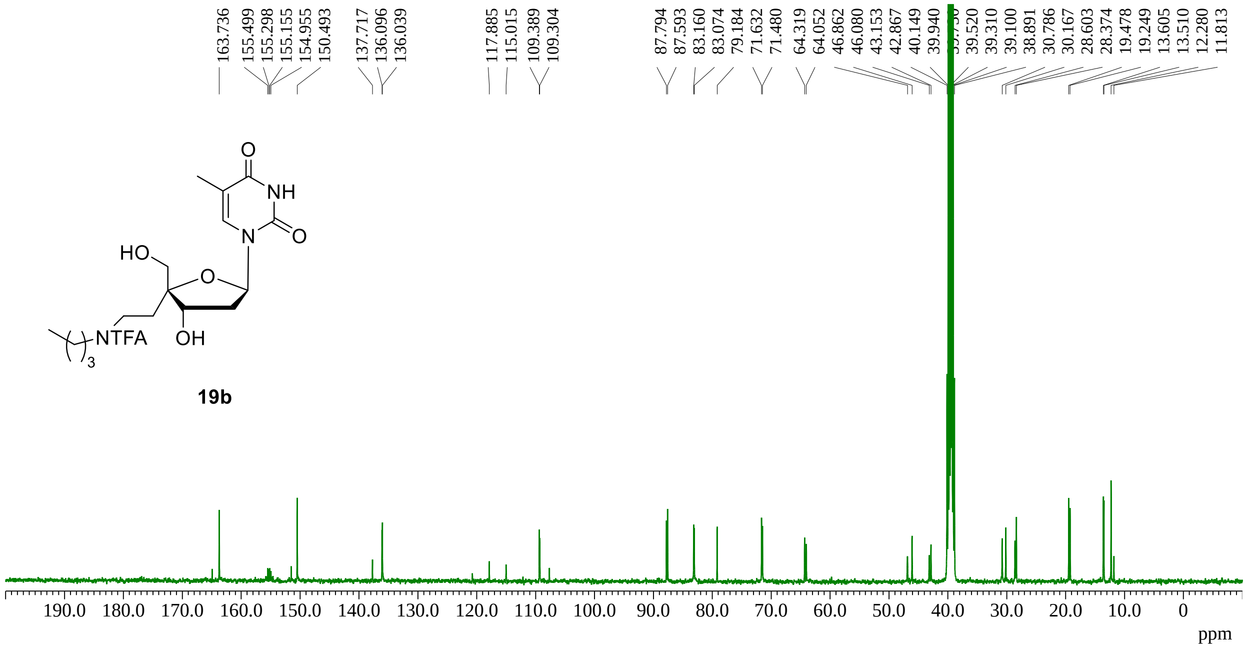

^{1}HNMR (400 MHz, DMSO-d_6) **19c**

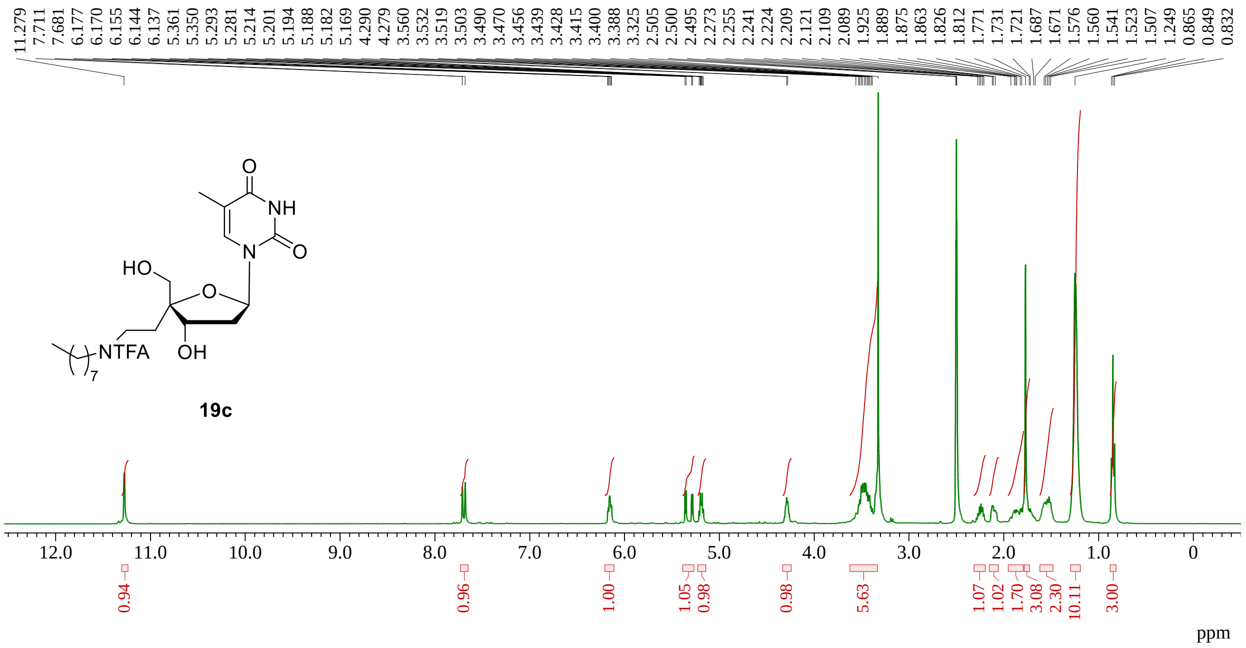

^{13}CNMR (101 MHz, DMSO-d_6) **19c**

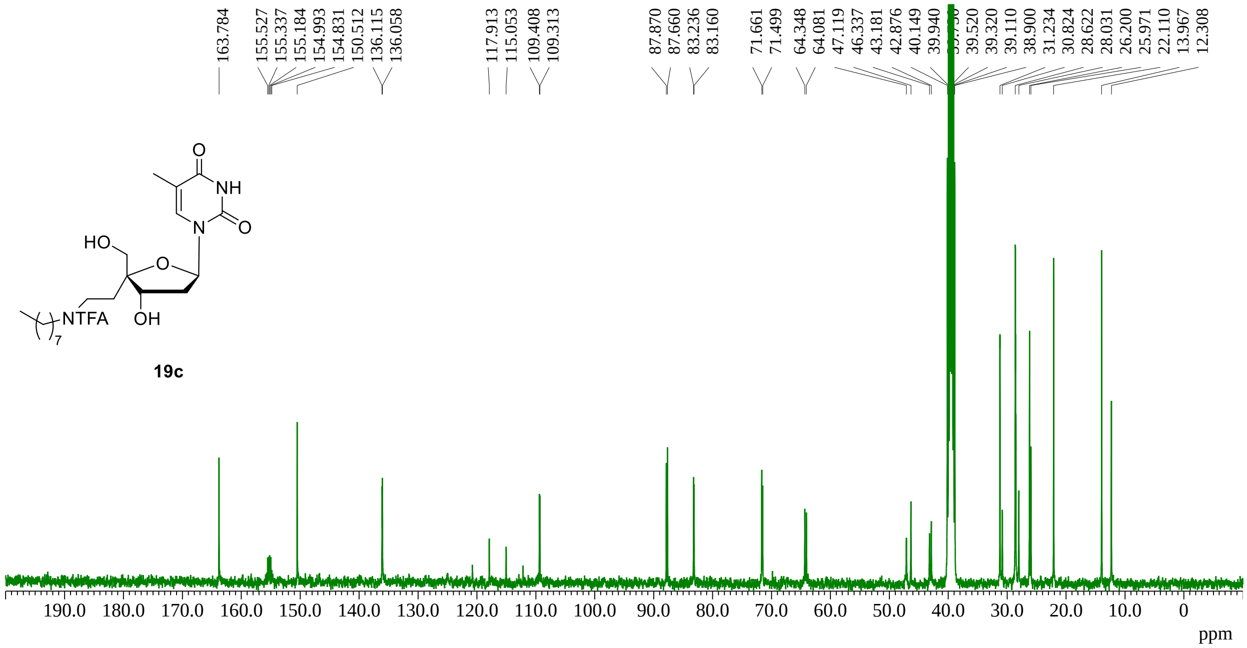

^{1}HNMR (400 MHz, $CDCl_3$) **20a**

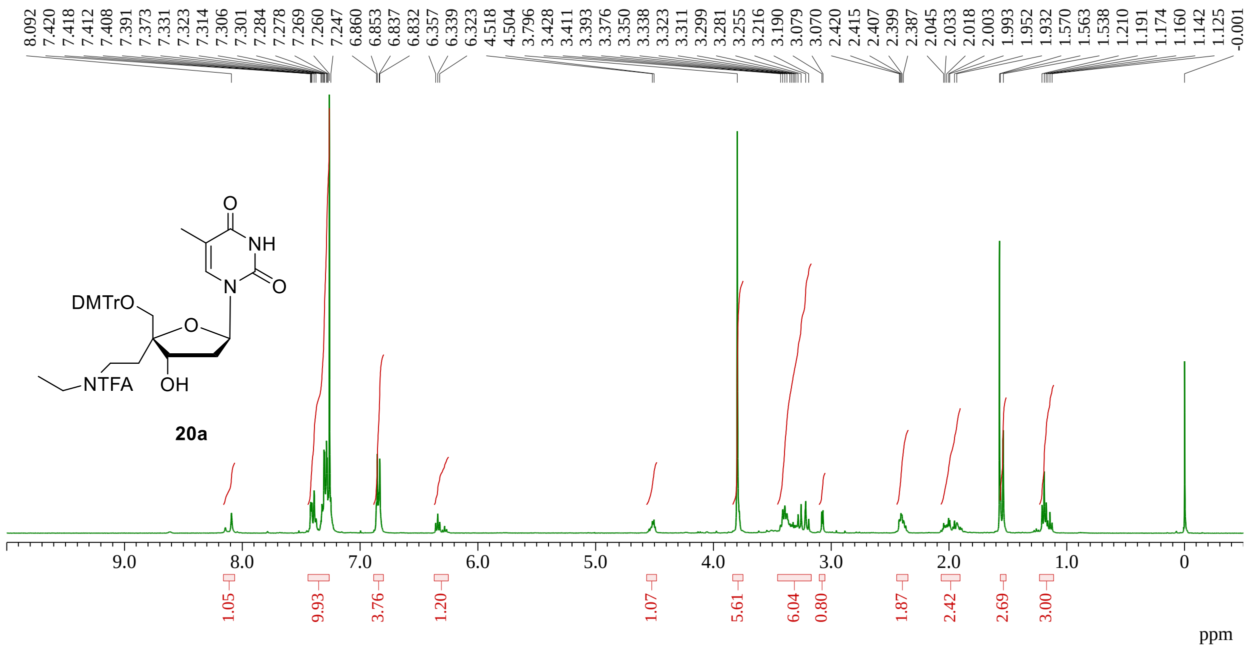

^{13}CNMR (101 MHz, $CDCl_3$) **20a**

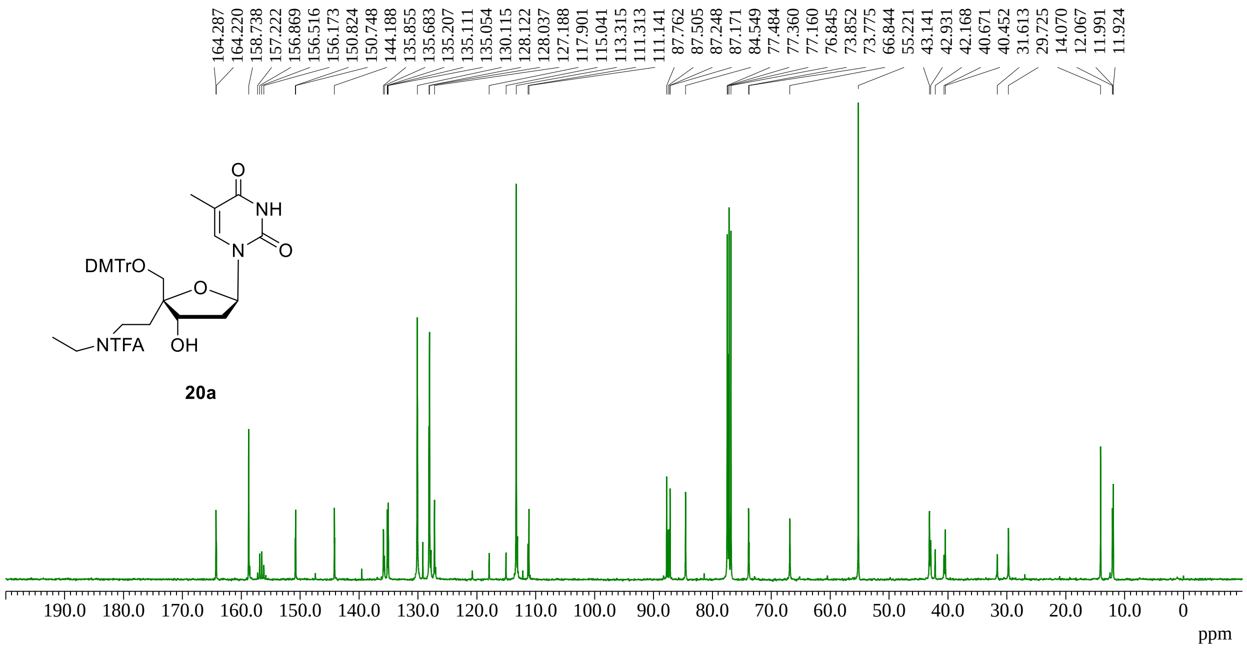

^{1}HNMR (400 MHz, $CDCl_3$) **20b**

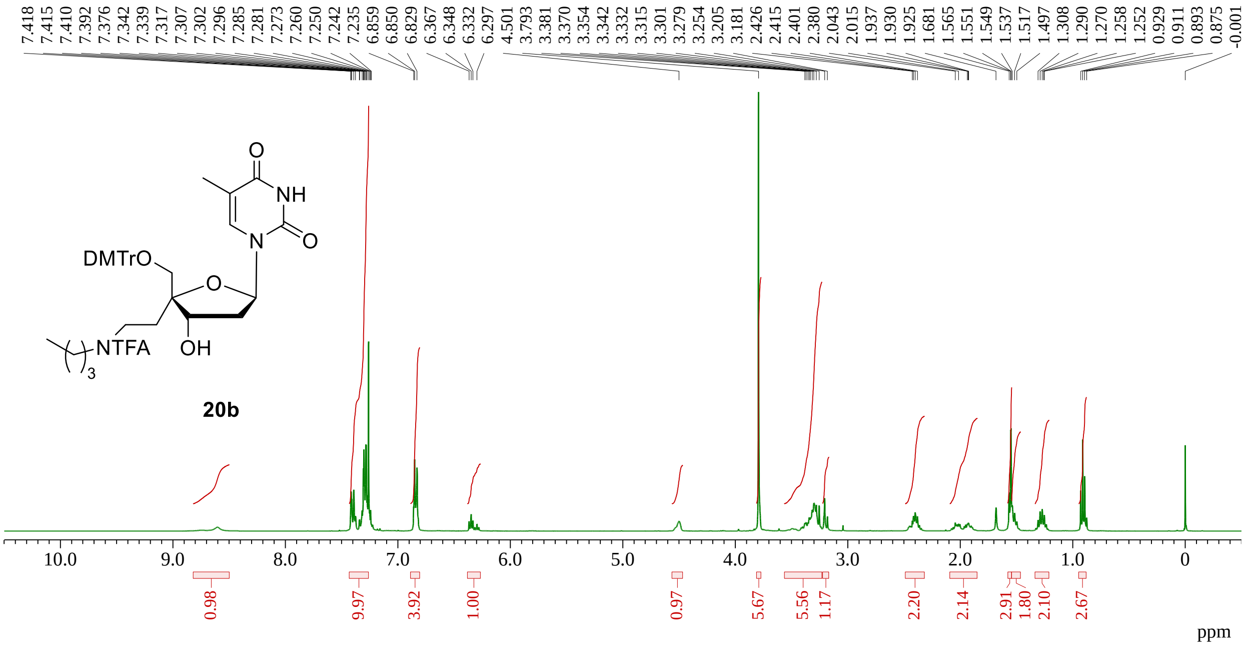

^{13}CNMR (101 MHz, $CDCl_3$) **20b**

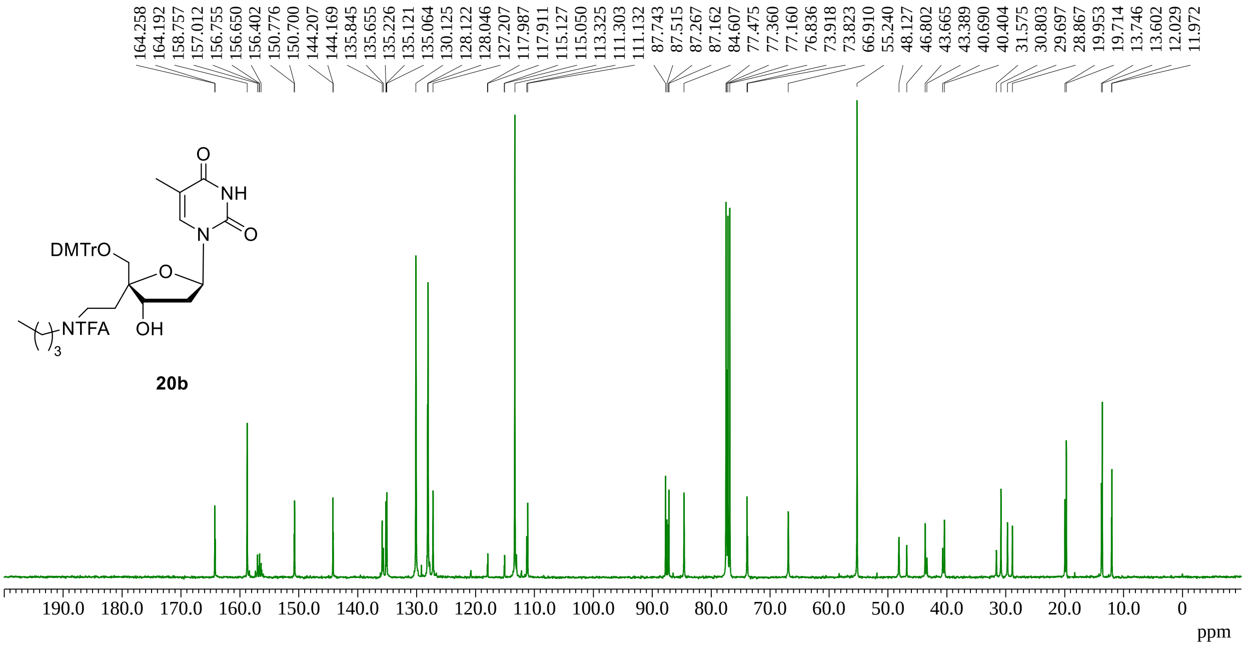

^{1}HNMR (400 MHz, $CDCl_3$) **20c**

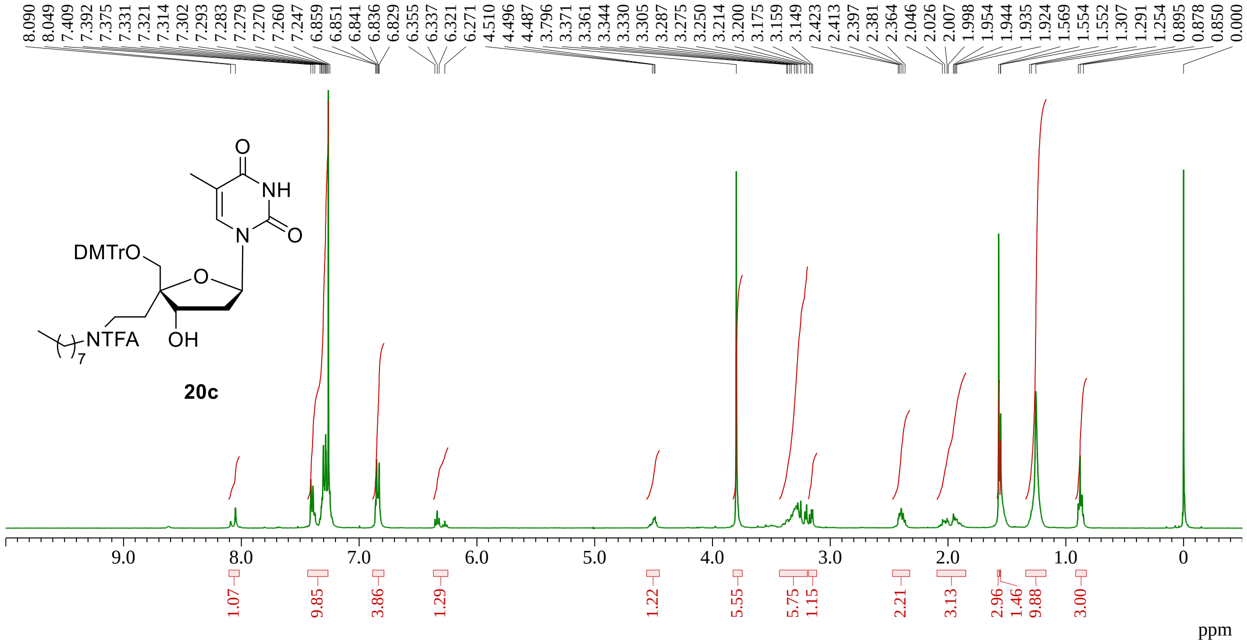

^{13}CNMR (101 MHz, $CDCl_3$) **20c**

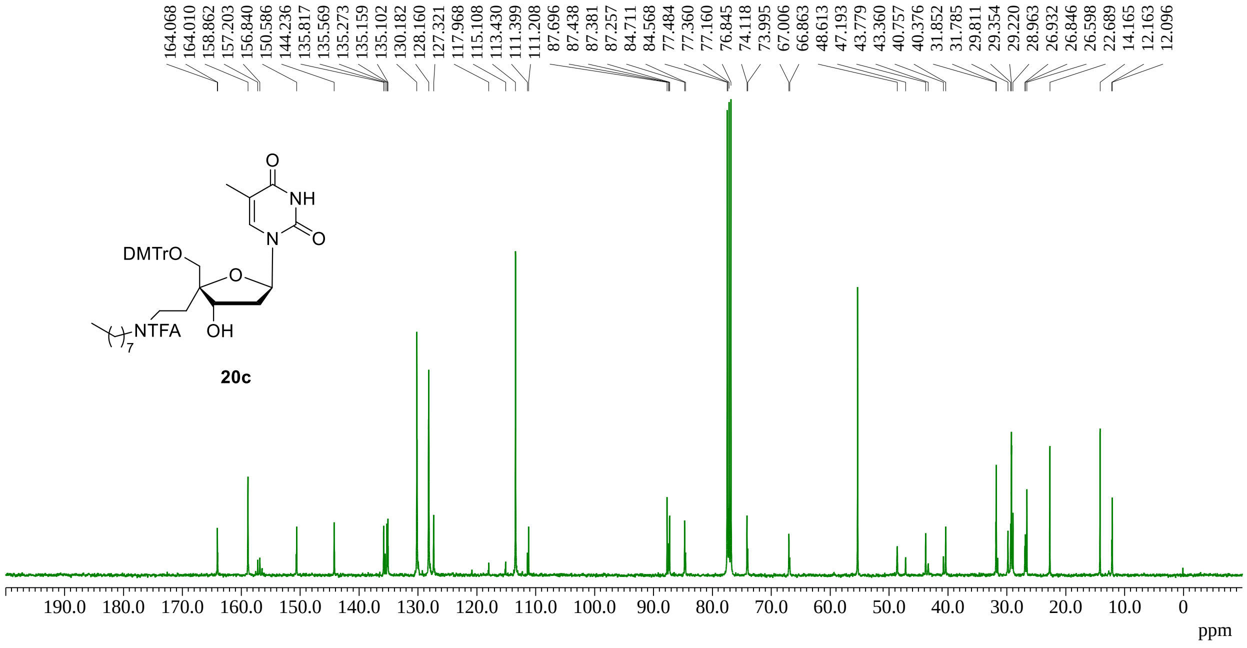

^{13}PNMR (162 MHz, $CDCl_3$) **21a**

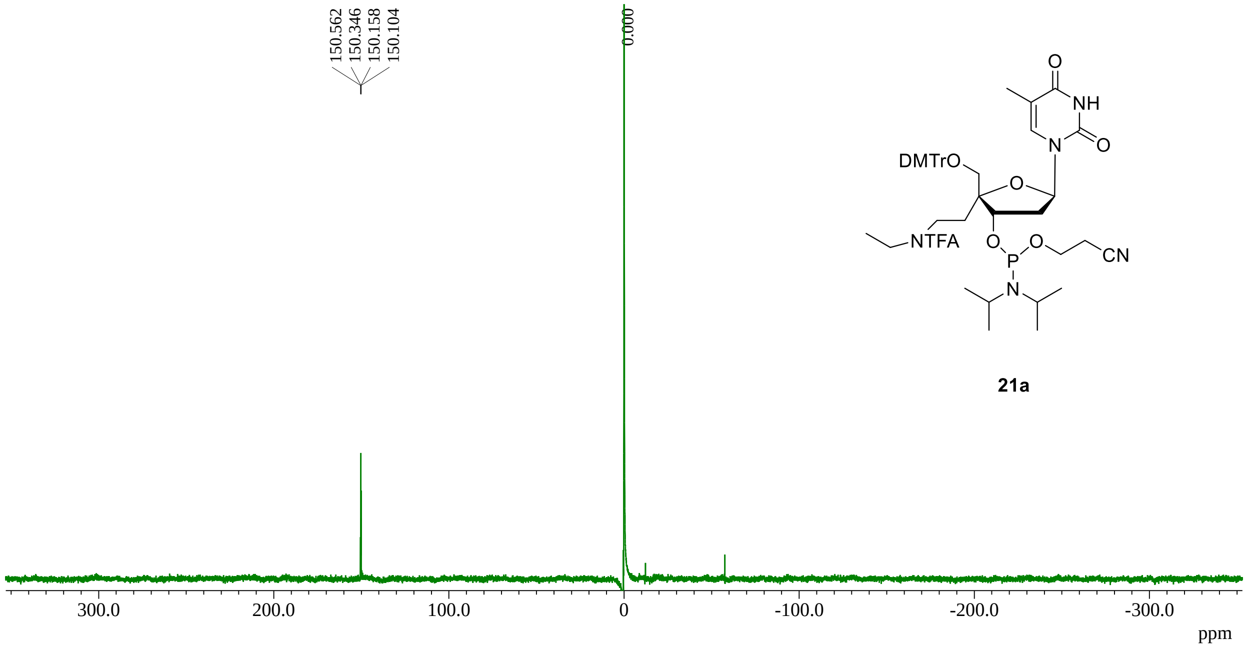

21a

^{13}PNMR (162 MHz, $CDCl_3$) **21b**

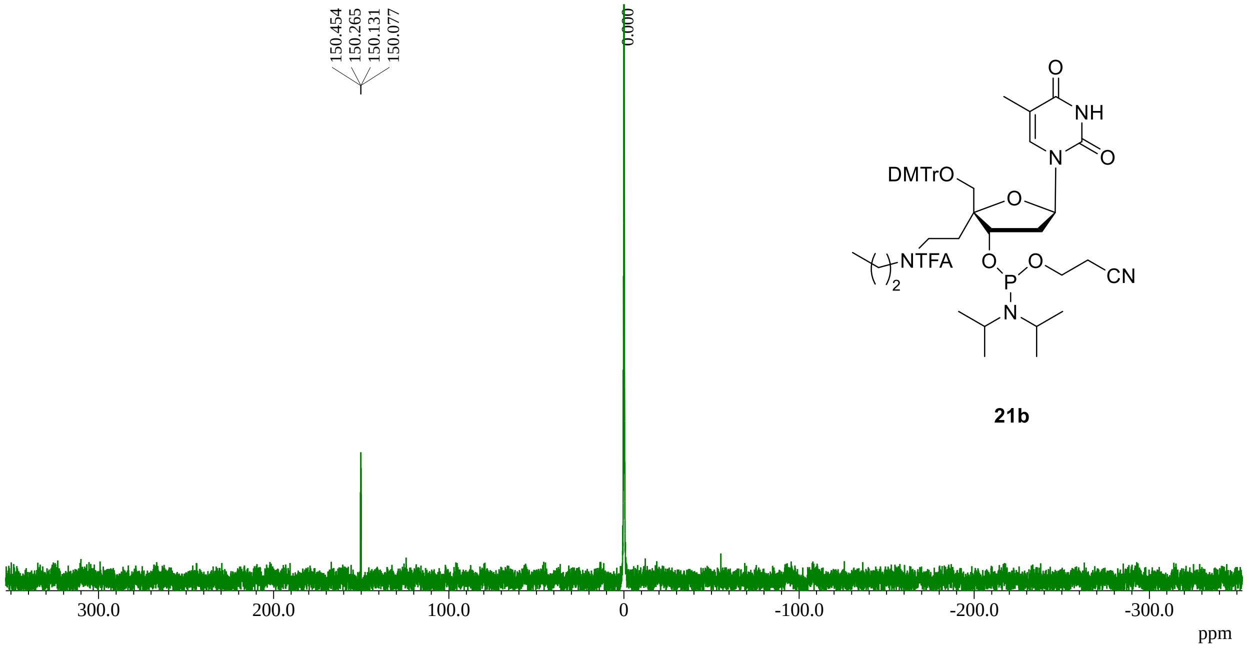

21b

^{13}PNMR (162 MHz, $CDCl_3$) **21c**

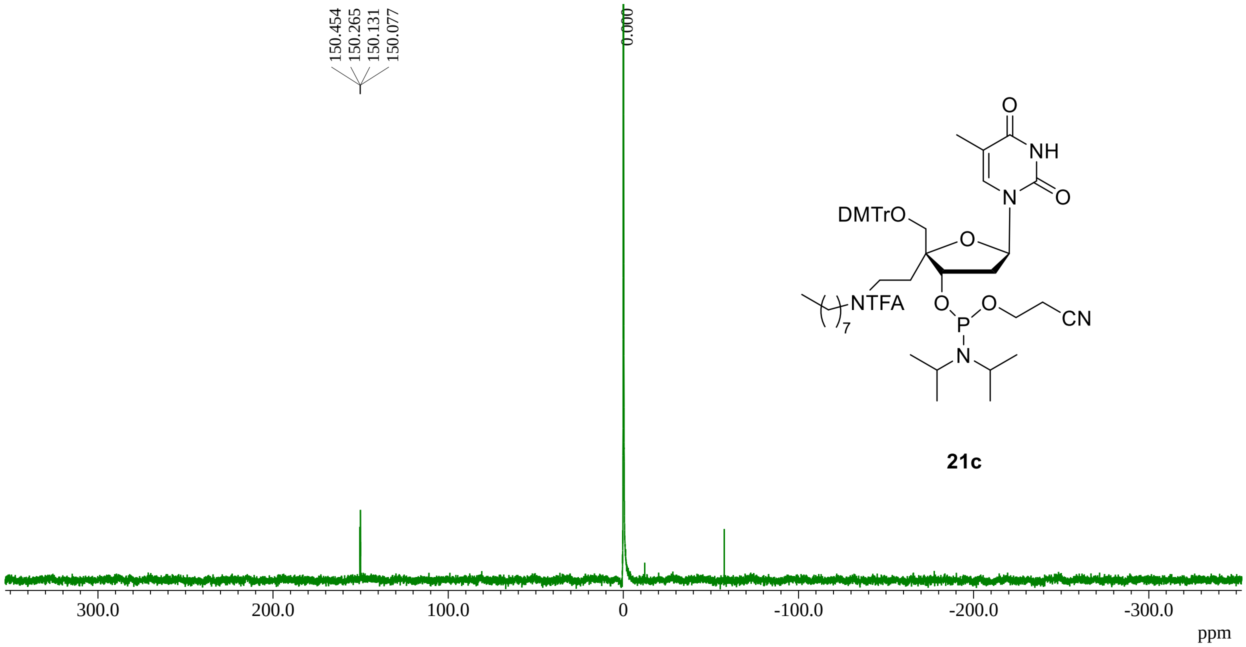

Supplement: Supplementary file 1 [file molecules-30-00581-s001.zip › molecules-3416354-supplementary.pdf]
